# Gap-com: general model selection criterion for sparse undirected gene networks with nontrivial community structure

**DOI:** 10.1093/g3journal/jkab437

**Published:** 2021-12-21

**Authors:** Markku Kuismin, Fatemeh Dodangeh, Mikko J Sillanpää

**Affiliations:** 1 Research Unit of Mathematical Sciences, University of Oulu, Oulu FI-90014, Finland; 2 Biocenter Oulu, University of Oulu, Oulu FI-90014, Finland; 3 School of Computing, University of Eastern Finland, Joensuu FI-80101, Finland; 4 Infotech Oulu, University of Oulu, Oulu FI-90014, Finland

**Keywords:** cluster, co-expression, complex network, gap statistic, high-dimensional data, model selection

## Abstract

We introduce a new model selection criterion for sparse complex gene network modeling where gene co-expression relationships are estimated from data. This is a novel formulation of the gap statistic and it can be used for the optimal choice of a regularization parameter in graphical models. Our criterion favors gene network structure which differs from a trivial gene interaction structure obtained totally at random. We call the criterion the gap-com statistic (gap community statistic). The idea of the gap-com statistic is to examine the difference between the observed and the expected counts of communities (clusters) where the expected counts are evaluated using either data permutations or reference graph (the Erdős-Rényi graph) resampling. The latter represents a trivial gene network structure determined by chance. We put emphasis on complex network inference because the structure of gene networks is usually nontrivial. For example, some of the genes can be clustered together or some genes can be hub genes. We evaluate the performance of the gap-com statistic in graphical model selection and compare its performance to some existing methods using simulated and real biological data examples.

## Introduction

Network modeling has been widely applied to examine the co-expression relationships between genes, which are estimated from the expression values using some co-expression measure (e.g. pairwise correlation between two genes). These measures indicate which genes are active simultaneously, implying that they are operating in the same biological processes (van Dam et al. 2018). In particular, gene clusters (groups of genes with a distinct pattern of pairwise co-expression relationships among each other) and hub genes (genes which are co-expressed with numerous other genes) have a key role in the functionality of the gene co-expression network itself (Barabasi and Oltvai 2004; Serin et al. 2016). Therefore, gene co-expression networks are usually complex and here they are assumed to be different from random networks, whose trivial community structure is determined by chance alone. For a review about methods which can be used for co-expression network modeling, see, e.g. Wang and Huang (2014). A special case of gene co-expression networks is obtained with the weighted gene co-expression analysis (WGCNA) (Zhang and Horvath 2005; Horvath 2011) which is a widely used technique. For some other highly cited articles (see, e.g. Stuart et al. 2003; Horvath and Dong 2008; Langfelder and Horvath 2008; Voineagu et al. 2011; Bailey et al. 2016).

As gene expression data sets are often high-dimensional, it is more useful to concentrate on examining only a much smaller subset of genes: we assume that most of the observed co-expressions between genes do not display relevant gene associations. Therefore, one can examine a smaller subset of significant gene associations directly by inducing sparsity into the estimated network topology resulting in a sparse network model. Several methods which can be applied in gene co-expression analysis, have been proposed for sparse network modeling. These methods are based on penalized estimation (Banerjee et al. 2008; Friedman et al. 2008), constrained optimization (Cai et al. 2011; Liu and Luo 2015; Liu and Wang 2017), and thresholding together with multiple testing correction (Ha and Sun 2014; van Wieringen and Peeters 2016) to mention a few examples (see also Rothman et al. 2008; Wang et al. 2016; Drton and Maathuis 2017; Kuismin and Sillanpää 2017). The common characteristic of these methods is that the sparsity of the output graph is usually controlled by a user-defined parameter (a tuning or a penalty parameter, a threshold parameter, a significance threshold, etc.) such that the model selection culminates in hyperparameter tuning. Here, we make the assumption that tuning of this single parameter is enough to recover all the pairwise co-expression relationships between genes in sufficient detail.

Despite the multitude of different model selection criteria such as the classic model selection criteria (CV, AIC, and BIC) (see, e.g. Bien and Tibshirani 2011; Liu et al. 2010) and model selection criteria developed especially for sparse networks such as the extended Bayesian information criterion (eBIC), GAIC and GBIC (Foygel and Drton 2010; Abbruzzo et al. 2019), and stability approach to regularization selection (StARS) (Liu et al. 2010) (see also Yuan and Lin 2007; Anandkumar et al. 2012; Vujačić et al. 2015; Kuismin and Sillanpää 2021), there are still only a few model selection methods available that evaluate different models based on the characteristics which are specific to complex networks (Mestres et al. 2018).

We are interested in finding nontrivial co-expression relationships, such as network communities (gene clusters) in our sparse network model when the network is constructed from gene expression values. In this study, we introduce the gap-com statistic (gap community statistic) that is a network model selection criterion used to estimate the quality of a collection of models for gene expression data. The gap-com statistic is used to choose biologically more relevant complex network models with distinguishable community structure. Our criterion shares characteristics with the gap statistic (Tibshirani et al. 2001) but there are a few features that distinguish the gap-com statistic from the original gap statistic: First, in the context of the network parameter estimation, one can control the number of clusters only indirectly, meaning that the number of clusters behaves like a random variable. Second, we introduce a new strategy that does not include data resampling but compares different models with sparse random network models constructed from rearranged data points. To the best of our knowledge, this kind of comparison has not been used with the gap statistic before.

Our formulation provides a basis for a general model selection technique for hyperparameter tuning, when the sparsity of the network model is controlled with one or more hyperparameters. We use simulated and empirical data sets to illustrate that the gap-com statistic is well suited for sparse network model selection, while the ground truth network has a community structure making it convenient for realistic gene co-expression analysis.

The paper is structured as follows. In Section Materials and methods, we describe the basic undirected network models applied in the rest of the paper and develop the gap-com statistic. Section Simulations provides simulation to evaluate the performance of the gap-com statistic in model selection. Section *Staphylococcus aureus* DREAM5 data illustrates the gap-com statistic with a real co-expression network analysis. Section Results and discussion concludes the paper with a discussion.

A demo script and R codes for reproducing all the analyses and figures represented in this study are available at GitHub under the GPL license, https://github.com/markkukuismin/gap-com.

## Materials and methods

### Sparse gene network construction

A data-driven gene co-expression network can be defined generally as follows: consider a multivariate random vector $Y={\left(Y_{1},Y_{2},\ldots ,Y_{p}\right)}^{⊤}$ where expression levels of genes *i* and *j* are denoted with two random variables *Y_i_* and *Y_j_*, respectively. The undirected graphical model G=(V,E), where V={i| i=1,…p} is a finite set of nodes (genes), and E⊆{(i,j)| i,j∈V,i≠j} is a set of the edges which correspond to gene-by-gene co-expression relationships. We emphasize that the definition “undirected” means that this model does not describe the direction of the co-expression relationship between genes. The graphical model **G** encodes the pairwise undirected co-expression relationship between all genes i=1,…p. In particular, the significant co-expression relationships between genes *i* and *j* are collected into the edge set **E** according to,

(1) (i,j)∈E⇔Yi ​⊥​​​⊥Yj,

where we use the “not independent” notation ​⊥​​​⊥ from probability theory to denote that there is a co-expression relationship between genes *i* and *j*. Usually, it is assumed that there are no self-loops in these gene networks; for each node *i*, (i,i)=0. Analyzing **G** is in general much easier than examining all possible pairwise co-expressions between genes one-by-one. In this article the terms “network” and “graph” have the same meaning.

The pairwise co-expression relationships between genes *i* and *j*, i,j=1,…,p, are usually unknown and must be estimated from the observed expression levels of *Y_i_* and *Y_j_* with some suitable measure. We use *n* to denote the number of these observations, commonly referred to as the number of samples. The co-expression network model can then be constructed from these gene-by-gene co-expression measures. In particular, two of the most widely used undirected graphical models applied in the co-expression analysis are Gaussian graphical models (GGMs) and models constructed using the Pearson correlation coefficients (hereafter called the correlation network) (Drton and Perlman 2007; Stuart et al. 2003). In GGMs, pair (*i*, *j*) is contained in the set **E** if and only if the partial correlation coefficient between genes *i* and *j* is measured as non-zero, that is, the correlation between genes *i* and *j* is non-zero given all other genes V∖{i,j} (Drton and Perlman 2007). In correlation network, pair (*i*, *j*) is contained in the set **E** if and only if the Pearson correlation coefficient between *i* and *j* is non-zero.

The difference between these models is that the Pearson correlation coefficient measures the linear co-expression relationships between genes and the partial correlation is capable of separating direct from undirect links between genes. Although the correlation might be sensitive to non-normality of the expression values, the measure itself does not assume normally distributed data like the partial correlation (GGMs) does. Both of these measures are unable to detect possible nonlinear co-expression relationships between genes. Other measures (coefficients) one can consider which are capable of modeling both linear and nonlinear dependencies between genes are, e.g. Spearman’s rank correlation, Kendall rank correlation coefficient, the distance correlation (Székely et al. 2007), mutual information (see, Husain et al. 2020, and the references therein), and the maximal information coefficient (Reshef et al. 2011). See also Kontio et al. (2020).

We make an assumption that the sparsity level of the undirected network of interest is low, meaning that $|E|/[(p^{2}-p)/2]\ll 1$, where |E| is the number of edges in the set **E** and $(p^{2}-p)/2$ is the maximum possible number of edges in the set **E**. Moreover, suppose that the complex network of interest has a clustering structure due to network communities or hubs. Assume that one can approximate the sparsity and the clustering structure of the ground-truth network by tuning just one external parameter of a network estimator used to construct the number of interactions among genes. To mention a few examples of these kind of methods, see the graphical LASSO (Glasso) algorithm (Friedman et al. 2008), Constrained *L*_1_-minimization for Inverse Matrix Estimation (CLIME) (Cai et al. 2011), and BIGQuic (Hsieh et al. 2013). We use the term “regularization parameter” and notation *λ* while referring to this parameter, which controls the sparsity of the network model for a given set of gene expression data. The data dependent fitted model of **G**, denoted here as $\hat{G}$, can be generically described as follows,

(2) $$\hat{G}=M_{\lambda }(Y),$$

where the estimator **M** depends on a parameter vector *λ*. In this study, we assume that *λ* is a positive scalar like, for example the tuning parameter related to the *L*_1_-norm in the Glasso algorithm in Friedman et al. (2008). Next, we describe how to tune this parameter while the presence of the community structure of the network is a preferable property. For simplicity, we will discuss about (partial) correlation while referring to the co-expression measure between a pair of genes (i,j),i,j=1,…p measured from the expression levels.

### The gap community statistic

Tibshirani et al. (2001) presented the original gap statistic in unsupervised clustering context to determine the number of clusters in a data set. Practical experience has shown that when one plots the within-cluster variation against the number of clusters, the variation first decreases rapidly, but after passing some value of the number of clusters, there is a visible deceleration of the decrease. Like Tibshirani et al. (2001) stated, statistical “folklore” informs that the location of such an “elbow” indicates the appropriate number of clusters. They proposed a rigorous method to choose the number of clusters by comparing the within-cluster variation with its expectation under the so-called reference distribution (data points with no distinct clustering structure). The estimate of *k* will be the value which maximizes the “gap” between observed within-cluster variation and its expectation under the reference distribution.

In unsupervised learning models, like in network construction, although the number of clusters (communities in a sparse network) cannot be controlled, the sparsity of the estimated network can be controlled by adjusting the value of a tuning parameter. By changing the sparsity level, one can indirectly influence how communities are determined by setting low co-expression measures (e.g. correlation coefficients which are close to zero) exactly to zero. In Kuismin et al. (2017), we noticed that if there is a distinct clustering structure in the genetic data, the community detection algorithm seems to favor a specific number of communities with certain values of the tuning parameter, although the sparsity of the corresponding network models change. We assume that the (community) structure of a data-driven gene co-expression network, which is close to the “true” clustering of the co-expression relationships between the genes, is more invariant to the changes of the parameters of the estimator **M** in (2) than a network where the co-expression relationship clustering structure is governed by noisy measurements. Therefore, instead of examining how the cluster dispersion measure deviates from the expectation under the reference distribution, we examine how the *number* of communities deviates from the expected number of communities under the null hypothesis that the pairwise co-expression relationships between genes are determined by chance. Thus, the constructed gene co-expression network does not show any clustering structure. Next, we propose how to approximate the number of communities under the null hypothesis by permuting the gene labels in the observed data.

Here, k(λ) denotes the number of communities depending on the regularization parameter *λ* evaluated with a community detection algorithm (these algorithms are discussed in more detail later in the article). We define our gap statistic as,

(3) $$\mathrm{Gap}(\lambda )=E\{k(\lambda )\}-\hat{k}(\lambda ),$$

where E{k(λ)} is the expected number of communities in the gene co-expression network model under the null hypothesis of random pairing of the gene co-expression relationships given regularization parameter *λ*. We use $\hat{k}(\lambda )$ to denote the number of communities inferred with some community detection algorithm from a data driven sparse network $\hat{G}$ depending on the value of *λ*. We aim to find the value of *λ* which maximizes the value of Gap(λ). In particular, we assume that the gene network corresponding to this maximizing value represents the complex structure of gene co-expression relationships in as exhaustive detail as we can detect from the gene expression values.

We propose 2 different strategies to estimate the unknown quantity E{k(λ)}. The first approach is based on permutations of the gene labels of the observed expression levels. We evaluate E{k(λ)} by repeating the permutation process several times and then compute the average of k(λ) over each random permutation. The workflow of this strategy is as follows:

1. For a single permutation of the observed data, randomly shuffle the gene expression values of each gene *i*, i=1,…p. Thus, the estimated pairwise (partial) correlation coefficients between genes *i* and *j* are determined randomly and we get the distribution of the co-expression relationships under the null hypothesis.
2. Construct the gene network from the permuted observed data with the regularization parameter value *λ* and identify the number of communities ${\hat{k}}_{n}(\lambda )$ in the network.
3. Repeat the first and the second step to evaluate the expected number of communities E{k(λ)} under the null hypothesis (i.e. communities are detected only by chance) as the mean value of the copies of ${\hat{k}}_{n}(\lambda )$.
4. Repeat steps 1–3 for each value of *λ*.

**Fig. 1:**
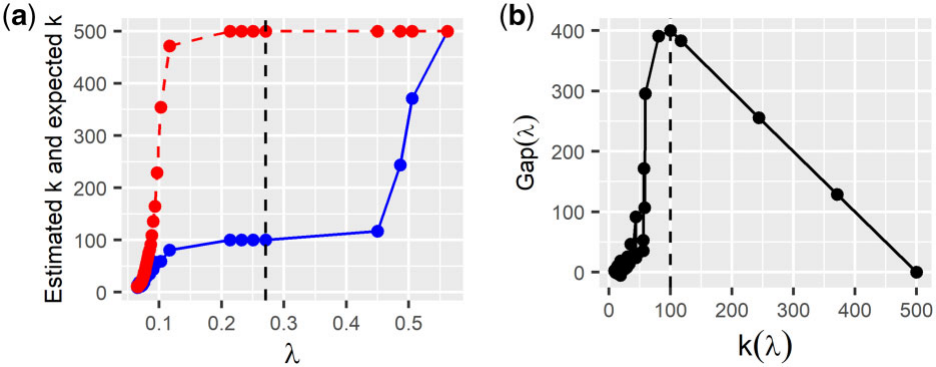
Permutation strategy. In this toy example, the ground truth graph is a star-graph with 100 distinct hubs (stars) (*p* = 500, *n* = 1,000). We use hard thresholding of pairwise correlation coefficients to compute sparse network estimates. a) Curves of estimated k(λ) (blue solid line) and estimator ${\hat{E}}_{n}\{k(\lambda )\}$ (red dashed line) are both determined with the Walktrap community detection algorithm as a function of the hard-threshold parameter *λ*. b) Gap-com curve as a function of estimated *k*. We set the number of permutations to 50 to estimate the unknown quantity E{k(λ)}. The vertical dashed line in the left-hand figure corresponds to the parameter value that maximizes Gap(λ). The vertical dashed line in the right-hand figure corresponds to the true number of hubs.

Define ${\hat{E}}_{n}\{k(\lambda )\}$ which denotes the estimator of the expected number of communities under the null hypothesis computed with the permutation process described above. We have illustrated the gap statistic computed using the permutation process in Fig. 1 where we use the Walktrap algorithm (Pons and Latapy 2006) to estimate the number of communities.

The second strategy is to apply the properties of the Erdős-Rényi random graph model G(p,s) (hereafter E-R graph). In the E-R model, a graph is constructed by connecting labeled genes randomly: Each gene-by-gene co-expression relationship is included in the gene co-expression network model with probability *s*, independently from other gene-by-gene co-expression relationships. The parameter *s* in this model can be considered as a weighting function: as *s* increases from 0 to 1, the model becomes more and more likely to become a dense graph and less and less likely to include sparse gene network structure. This is why *s* can be affected by altering the tuning parameter *λ*. The workflow of our second strategy is as follows:

1. Permute the observed expression levels of all genes once.
2. Construct the gene network from the permuted expression level data from the first step with the regularization parameter value *λ*.
3. Estimate the probability *s* with the sparsity level of the constructed network depending on the value of the regularization parameter *λ*. The sparsity level *s* can be computed by dividing the number of edges of the graph by the maximum possible number of edges $(p^{2}-p)/2$. We denote this estimation with $\hat{s}(\lambda )$.
4. Generate copies of the E-R graph $G(p,\hat{s}(\lambda ))$ and identify the number of communities ${\hat{k}}_{G}(\lambda )$ in each copy to evaluate the expected number of communities E{k(λ)}, detected by chance, as the mean value of the copies of ${\hat{k}}_{G}(\lambda )$.
5. Repeat steps 1–4 for each value of *λ*.

**Fig. 2.**
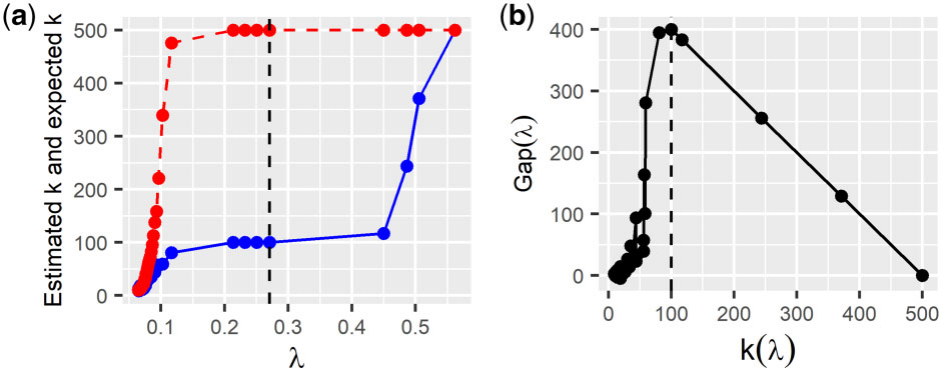
E-R model strategy. In this toy example, the ground truth graph is a star-graph with 100 distinct hubs (stars) (*p* = 500, *n* = 1,000). We use hard thresholding of pairwise correlation coefficients to compute sparse network estimates. a) Curves of k(λ) (blue solid line) and reference ${\hat{E}}_{G}\{k(\lambda )\}$ (red dashed line) are both determined with the Walktrap community detection algorithm as a function of the hard-threshold parameter *λ*. b) Gap-com curve as a function of estimated *k*. We generated 50 copies of the E-R graph to estimate E{k(λ)}. The vertical dashed line in the left-hand figure corresponds to the parameter value that maximizes Gap(λ). The vertical dashed line in the right-hand figure corresponds to the true number of hubs.

**Fig. 3.**
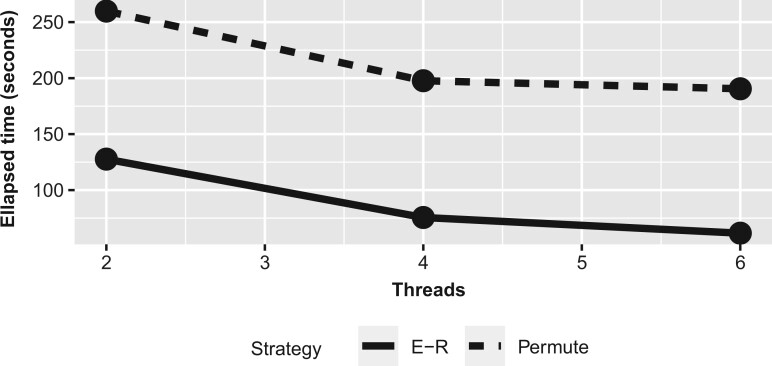
The elapsed times of both resampling strategies as a function of parallel threads. We use the hard thresholding of pairwise correlation coefficients to compute the sparse network estimates (*p* = 1,000, *n* = 200). We set the number of permutations to 50 in the first strategy and likewise generated 50 copies of the E-R graphs to evaluate the gap-com statistic.

We define ${\hat{E}}_{G}\{k(\lambda )\}$ as the estimator of the expected number of communities under the null hypothesis computed with the E-R graph resampling process, where the edge inclusion probability is equal to the sparsity level s(λ). Hence, instead of finding the number of communities under the null hypothesis using data permutations, we simulate E-R graphs which are much faster. Note that we propose to use just one permutation of the data to estimate the parameter *s* to ease the computational complexity of the second strategy. The simulation results presented in the next section indicate that the gap-com statistics computed with the first and the second strategy are practically the same. Therefore, it seems that just steps 4 and 5 of the second strategy need to be repeated. This decreases the time complexity of the second strategy substantially. See also Fig. 2 where we illustrate the gap-com statistic computed applying the E-R model strategy. As one can see, both Figs. 1 and 2 are almost identical and Gap(λ) is maximized with the same threshold parameter. This suggests that both strategies are very similar while the second one is faster than the first strategy. However, it is easy to mitigate the computational expense of both of the strategies with parallel computing. We have illustrated how parallel computing can reduce the time needed to compute the gap-com model selection criterion in Fig. 3.

We choose *λ* which maximizes Gap(λ) as presented in (3). The tuning parameter chosen with the gap-com statistic has an easily understandable interpretation: the selected sparse graphical model reflects the complex clustering structure of the gene co-expression network which differs from the co-expression network whose pairwise gene-by-gene relationships are determined just by chance.

The value of the regularization parameter *λ*, which maximizes Gap(λ), might not be unique if the data strongly supports a specific number of clusters (Kuismin et al. 2017). However, the network models which maximizes Gap(λ) with different values of *λ* are usually not isomorphic. We do not want to miss interesting network characteristics (hubs and clusters) but we still want to reduce the number of false-positive edges (co-expression relationships which are estimated as significant but are not so in reality). In such case, we choose the regularization parameter value which produces a network model with the smallest sparsity level (the simplest model principle/parsimony principle), more generally: choose the tuning parameter value *λ* which produces the simplest network model such that $\mathrm{Gap}(\lambda )\geq \mathrm{Gap}(\lambda^{\prime }),\;\lambda \neq \lambda^{\prime }$.

In addition to the tuning parameter *λ*, the gap-com statistic depends on the community detection algorithm used to find the number of communities (*k*). For the sake of clarity, we use just one community detection method to illustrate the gap-com statistic. In particular, we use the Walktrap algorithm with the gap-com statistic to determine the number of clusters in a constructed network. Walktrap uses random walks and encodes results of the walks into a dendrogram. Then one can use the modularity score to decide the optimal community structure of the network (the cutoff of the dendrogram). Although the Walktrap algorithm is computationally quite expensive, it has been shown that it correspond closely to the ground truth communities (Danon et al. 2005; Yang et al. 2016) similar to the performance of the multilevel algorithm which is not discussed in this study (Blondel et al. 2008).

### Simulations

To illustrate the performance of gap-com in graphical model selection, we generate artificial test data from a multivariate Gaussian distribution with a zero mean vector and a precision matrix whose sparsity structure depends on 3 different undirected complex network models, and one random model without a clear clustering structure. The simulation models are the following:

1. A *cluster-graph* with 10 disjoint clusters.
2. A *star-graph* with 10 disjoint stars.
3. The Barabási–Albert network model (or shortly the random *scale-free graph*).
4. The E-R network model G(p,s=0.263) (or shortly as *random graph*).

We examine the case where *p* = 500.

This cluster-graph consists of 10 disjoint E-R networks G(p,s=0.3) where the probability parameter *s* is set as 0.3 which is the default value of the R-package huge (version 1.3.2) (Zhao et al. 2012). The Barabási–Albert model does not generate disconnected clusters leading to ambiguous community detection. Nevertheless, it is an important class of complex networks, so we use it to illustrate how the gap-com statistic works when the underlying complex network shows no clear clustering structure. On the other hand, the E-R graph is known to show very low or no clustering at all. Although this graphical model might not represent real-world gene co-expression network structure we use it in this simulation example to illustrate that the model selection criteria do force clustering structure into the network model in case there is no clear clustering structure present. In the E-R graph model G(p,s), we set the parameter *s* as 0.263. We choose this value experimentally. The graph generated with this value has just a few clusters detected with the Walktrap algorithm in our simulations. In particular, we detected 5 unique communities with the Walktrap algorithm in the graph simulated with the E-R graph model.

As mentioned earlier, test data sets are generated under the complex network models described above using the R package huge. We use *n* = 200 samples to estimate the sparsity pattern of precision and correlation matrices used to construct different networks (ratio *n*/*p* is equal to 0.4). The number of features commonly exceeds the number of observations by far in high-dimensional gene co-expression relationship estimation. For example, in Schäfer and Strimmer (2005) authors examined a breast cancer data set with 3,883 genes and 49 samples (n/p=0.0130) (West et al. 2001). We have set the sample size so that we obtain comparable results with all the model selection methods (see below).

We use 2 methods to estimate the pairwise relationships of the simulated random variables: hard thresholding (see, e.g. Horvath 2011) and BigQuic (hereafter BQ) (Hsieh et al. 2013). We choose these methods because they are the most suitable ones for large-scale (say, tens of thousands of nodes) network construction. Moreover, hard thresholding represents a correlation network construction tool and BQ a GGM construction tool. Both methods are publicly available in the Comprehensive R Archive Network (CRAN) and implemented in packages huge and BigQuic. We use 50 different regularization parameter values for both methods. In addition, we restrict the largest sparsity level of the network models considered here to 0.1 when the hard-thresholding is used to construct the sparse network from simulated data.

To illustrate the gap-com statistic, we generate 50 data permutations and 50 copies of the E-R graph to evaluate E{k(λ)} under the null hypothesis while using the Walktrap algorithm to detect clusters in a network model. We call the gap-com statistic utilizing data permutation strategy and the gap-com statistic computed using E-R graph resampling strategy “gap-com (perm)” and “gap-com (E-R),” respectively. We set the length of the random walk to four for the Walktrap algorithm and choose the optimal number of communities based on the highest modularity score.

For comparison, we also performed graphical model selection using three other model selection criteria to compare these graphical models with the graphical models selected with the gap-com statistic: StARS (Liu et al. 2010), path connectivity (PC), and AGlommerative NESted (AGNES) (Mestres et al. 2018). StARS is also based on resampling of the observed data and we thus generated 50 subsamples for StARS. StARS incorporates an extra tuning parameter to control the sparsity of the selected sparse network. We set it as 0.05 which is the default value proposed by Liu et al. (2010). PC compares the change between geodesic distance mean statistics of 2 consecutive sparse models (corresponding to 2 consecutive tuning parameter values). PC criterion chooses the model where the change in graph complexity is at its peak and this is like the gap statistic, but PC is not based on resampling. AGNES chooses the graphical model which maximizes an agglomeration coefficient (AC). We note that the above-mentioned graphical model selection criteria are general methods which work with any network estimator. In this illustrative example, our strategy is to choose the “best” model among the same collection of sparse network models. Thus, 2 different criteria can select the same sparse network model.

While comparing these 5 model selection criteria with each other, we wanted to examine how accurately the selected edges reflect the structure of the underlying true complex graphical model and how the estimated communities correspond to the known community structure computed from the ground truth network with the Walktrap algorithm.

We use the following binary classification metrics to compare the selected network model edge-wise to the ground truth network:where *TN* is the number of true negatives, *FP* is the number of false positives, *TP* is the number of true positives and *FN* is the number of false negatives. We define *Pre* = 0 and *MCC* = 0 if TP+FP=0. Sensitivity and precision can vary between 0 and 1 and MCC between –1 and 1. The closer the values of Sen, Pre and MCC are to one, the better the model performance is.

1. Sensitivity Sen =TP/(TP+FN).
2. Precision Pre =TP/(TP+FP).
3. Mathew correlation coefficient MCC $=\gamma \times \beta^{-0.5}$, where γ=(TP×TN−FP×FN) and β=(TP+FP)×(TP+FN)×(TN+FP)×(TN+FN),

To compare the detected communities with the ground truth, we compute the normalized mutual information (NMI) measure formalized, e.g. in Danon et al. (2005); Kuncheva and Hadjitodorov (2004). Assume that *A_w_* and *B_w_* are communities of the ground truth and the selected graphical model derived with the Walktrap algorithm respectively. Then the NMI between these clusters is,

(4) $$\mathrm{NMI}(A_{w},B_{w})=\frac{-2\sum_{i=1}^{C_{A}}\sum_{j=1}^{C_{B}}p_{ij}\;\text{log}(p_{ij}p/p_{.i}p_{j.})}{\sum_{i=1}^{C_{A}}p_{i.}\;\text{log}(p_{i.}/p)+\sum_{j=1}^{C_{B}}p_{.j}\;\text{log}(p_{.j}/p)},$$

where *C_A_* and *C_B_* are the numbers of clusters in *A_w_* and *B_w_*, respectively (*C_A_* and *C_B_* do not need to be equal), *p_ij_* is the number of nodes in both the cluster *i* of partition *A_w_* and the cluster *j* in partition *B_w_*, and $p_{i.}$ and $p_{.j}$ are the numbers of nodes in clusters *i* and *j* of partitions *A_w_* and *B_w_* respectively. NMI values are between zero and one. If the communities of the selected and the ground truth graph are identical, then NMI is equal to one. If the selected and ground truth communities are independent, then NMI is equal to zero.

We report the results of Sen, Pre, MCC, and NMI in Tables 1 and 2 averaged over 100 simulation replications. We also report the average number (median) and the interquartile range (IQR) of the number of unique communities the Walktrap algorithm identified from each selected network model. Because both the hard thresholding and BigQuic return a very sparse support for the graphical models, we removed isolated nodes (nodes with zero degree) from the graphical model selected with gap-com, StARS, PC, and AGNES to ease the interpretation of the results. We denote the number of identified communities after removal of isolated nodes with “# Clusters.”

**Table 1.** Averaged model evaluation metrics of binary classification tests (mean), NMI (mean), and the number of clusters [median (IQR) after isolated nodes are removed] over 100 replications from different sparse network models while using hard thresholding (Threshold) when *p* = 500 and *n* = 200.

Graph model	Method	Criterion	Sen	Pre	MCC	NMI	No. of clusters
Cluster	Threshold	gap-com (perm)	0.68	0.17	0.33	0.75	16 (4.00)
Cluster	Threshold	gap-com (E-R)	0.68	0.17	0.33	0.75	16 (4.00)
Cluster	Threshold	StARS	0.57	**0.27**	**0.38**	**0.82**	11 (1.00)
Cluster	Threshold	PC	**0.70**	0.14	0.31	0.73	17 (4.00)
Cluster	Threshold	AGNES	0.66	0.19	0.34	0.76	15 (4.00)
Star	Threshold	gap-com (perm)	0.70	0.80	0.74	0.75	10 (0.00)
Star	Threshold	gap-com (E-R)	0.69	0.80	0.74	0.75	10 (0.00)
Star	Threshold	StARS	0.37	**0.92**	0.58	0.65	10.50 (1.00)
Star	Threshold	PC	**0.74**	0.77	**0.75**	**0.76**	10 (0.00)
Star	Threshold	AGNES	0.52	0.87	0.67	0.69	10 (0.25)
Scale-free	Threshold	gap-com (perm)	**0.43**	0.18	**0.27**	**0.63**	29 (17.25)
Scale-free	Threshold	gap-com (E-R)	0.41	0.19	**0.27**	0.62	30 (12.75)
Scale-free	Threshold	StARS	0.22	**0.27**	0.24	0.50	54 (12.00)
Scale-free	Threshold	PC	0.24	0.26	0.25	0.52	50 (13.00)
Scale-free	Threshold	AGNES	0.23	**0.27**	0.24	0.51	52 (12.25)
Random	Threshold	gap-com (perm)	0.53	**0.02**	**0.06**	0.17	55 (15.00)
Random	Threshold	gap-com (E-R)	0.53	**0.02**	**0.06**	0.17	55 (14.25)
Random	Threshold	StARS	0.52	**0.02**	**0.06**	0.18	57.5 (16.00)
Random	Threshold	PC	**0.55**	0.01	0.05	**0.22**	45 (11.25)
Random	Threshold	AGNES	0.54	0.01	0.05	0.21	47.5 (12.00)

The highest averaged value are boldfaced in each column. The true numbers of communities are 10, 10, 40, and 5 of the cluster, star, scale-free, and the random graph model, respectively.

**Table 2.** Averaged model evaluation metrics of binary classification tests (mean), NMI (mean), and the number of clusters [median (IQR) after isolated nodes are removed] over 100 replications from different sparse network models while using BigQuic (BQ) when *p* = 500 and *n* = 200.

Graph model	Method	Criterion	Sen	Pre	MCC	NMI	No. of clusters
Cluster	BQ	gap-com (perm)	0.68	0.17	0.33	0.75	16 (3.25)
Cluster	BQ	gap-com (E-R)	0.68	0.17	0.33	0.75	16 (5.00)
Cluster	BQ	StARS	0.53	**0.29**	**0.38**	**0.83**	10 (1.00)
Cluster	BQ	PC	**0.70**	0.14	0.30	0.72	18 (4.00)
Cluster	BQ	AGNES	0.65	0.19	0.34	0.76	15 (3.50)
Star	BQ	gap-com (perm)	0.60	0.80	0.68	**0.76**	10 (0.00)
Star	BQ	gap-com (E-R)	0.59	0.80	0.68	0.75	10 (0.00)
Star	BQ	StARS	**0.81**	0.72	**0.76**	0.75	10 (0.00)
Star	BQ	PC	0.63	0.78	0.69	0.75	10 (0.00)
Star	BQ	AGNES	0.40	**0.86**	0.57	0.70	10 (1.00)
Scale-free	BQ	gap-com (perm)	0.41	0.18	**0.27**	**0.63**	26.50 (12.00)
Scale-free	BQ	gap-com (E-R)	**0.42**	0.18	**0.27**	**0.63**	27 (12.25)
Scale-free	BQ	StARS	0.13	**0.37**	0.20	0.37	48 (27.00)
Scale-free	BQ	PC	0.36	0.21	0.24	0.57	43 (31.25)
Scale-free	BQ	AGNES	0.17	0.26	0.20	0.52	53 (18.00)
Random	BQ	gap-com (perm)	0.53	0.02	0.06	0.18	55 (22.00)
Random	BQ	gap-com (E-R)	0.53	0.02	0.06	0.18	54.50 (23.25)
Random	BQ	StARS	0.48	**0.04**	**0.07**	0.13	53 (23.50)
Random	BQ	PC	**0.54**	0.01	0.05	**0.21**	49 (18.25)
Random	BQ	AGNES	0.50	**0.04**	0.05	0.20	50 (16.25)

The highest averaged value are boldfaced in each column. The true numbers of communities are 10, 10, 40, and 5 of the cluster, star, scale-free, and the random graph model, respectively.

### *Staphylococcus aureus* DREAM5 data

Here, we infer a co-expression network from an expression data set of the human pathogen *Staphylococcus aureus* (hereafter *S. aureus*) used previously in the DREAM5 (Dialogue on Reverse Engineering Assessment and Methods) network inference challenge (Marbach et al. 2012). Overall, there are expression levels of 2,810 genes for 160 samples. From these 2,810 genes, about 5% are decoy genes introduced by randomly selecting gene expression values from the compendium itself (see Supplementary Note 1 of Marbach et al. 2012). Genes were anonymized for the original DREAM5 challenge, but true gene IDs are available on the DREAM Challenges homepage. The underlying network is unknown, but we assume that the genes interact with each other so that they form nontrivial co-expression relationships. There are in total 99 potential transcription factors (TFs) from which 9 are decoy genes (false positives). The expression data have been uniformly normalized.

We note that in the original work of Marbach et al. (2012) authors examined regulatory interactions between genes (gene regulatory networks) and we examine a co-expression network. However, these 2 network classes could somehow be related (Xulvi-Brunet and Li 2010). Here we briefly compare these networks with each other. In particular, we inspect a network community which corresponds to a module of 27 genes identified in Marbach et al. (2012) that is highly enriched for pathogenic genes. Hereafter, we refer to this community as Pathogen module.

We use hard-thresholding of pairwise correlation coefficients to control the sparsity of the gene co-expression network and test 50 different hard-threshold cutoff values in the co-expression network construction. We restrict the largest sparsity level of the gene co-expression network models considered here to 0.3. We again apply the gap-com statistic (the E-R strategy) in graphical model selection and StARS, PC, and AGNES for comparison. Again, we use the Walktrap algorithm to identify clusters from the constructed network models and set the length of the random walk in the Walktrap algorithm to 4. We simulate 100 E-R graphs while applying the gap-com statistic (the second strategy) and 100 subsamples while utilizing StARS. We set the extra tuning parameter of StARS criterion to 0.2. As pre-processing, we modify the gene expression levels using the nonparanormal transformation (Liu et al. 2012), also provided in the R package huge, to ease the assumption of normality in our analysis.

In addition to the pathogen community, we examined how the inferred hub nodes correspond to genes that are potential TFs for the *S.aureus* network. We list the first 100 hub nodes, which correspond to the set of the 99 given TFs in Table 3. We also examined the most connected hub nodes (hub genes). We have reported the first 10 of the most connected genes in Table 4.

**Table 3.** The first of the 100 most connected nodes which correspond to potential TFs of the DREAM5 *S. aureus* network.

Criterion	TFs
gap-com	SAV1228
StARS	None
PC	SAV0044
AGNES	SAV1228 SAV1686, SAV2046

**Table 4.** The first 10 of the most connected genes (nonzero degree) according to their degrees in the DREAM5 *S. aureus* networks by the model selection criterion.

gap-com		PC		AGNES	
Gene ID	Degree	Gene ID	Degree	Gene ID	Degree
SAV0996	915	SAV1355	152	SAV1215	1,524
SAV1215	911	SAV1596	135	SAV0996	1,498
SAV2347	911	SAV2596	130	SAV0568	1,482
SAV0669	903	SAV0398	127	SAV0669	1,477
SAV0952	901	SAV0537	124	SAV1192	1,462
SAV0014	893	SAV0069	124	SAV2071	1,442
SAV1892	890	SACOL0041	123	SAV2347	1,442
SAV1900	890	SACOL0054	122	SAV0602	1,438
SAV0654	889	SAV0400	121	SAV0390	1,432
SAV2071	871	SAV0407	120	SAV2221	1,425

The graph selected with StARS is left out because all nodes in the graph had a degree of zero.

## Results and discussion

### Simulation results

The averaged simulation results of both gap-com strategies are practically identical. The E-R model strategy seems to be an efficient way to compute the gap-com statistic in terms of model recovery and computational cost. We also collected the tuning parameter values selected in each simulation round. The values selected with both gap-com strategies were almost identical. On the other hand, the tuning parameters selected with different model selection criteria were somewhat different on average (see the Supplementary Materials).

Remarkably, the network model selected using the gap-com statistic returns the highest averaged NMI estimates when the ground truth network is generated using the Barabási–Albert model (scale-free network). Except for this, there is no striking difference between the model selection criteria used in this example. For example, the median number of communities identified from the data constructed using the star-graph was exactly ten for all the methods after the isolated nodes were removed from the selected graphs. All criteria have difficulties to detect the structure of the E-R model. For example, the averaged values of precision and MCC are really low (see Tables 1 and 2). We recall that the model selected with StARS depends on an extra tuning parameter and this hyperparameter is usually not tuned during the StARS process. The gap-com statistic, PC and AGNES can estimate the quality of a collection of network models over several tuning parameters during the model selection process.

To test how the methods are affected when there are “orphan nodes” (nodes with zero degree) in the ground truth network, we repeated the previous simulation example but this time we removed 25% of the edges in the ground truth network models at random. In this re-run, we used just the hard-thresholding and 50 simulation replications. When there are orphan nodes in the ground truth network, the averaged NMI values associated with gap-com strategies and PC increased and are very similar. Moreover, averaged NMI values associated with gap-com strategies and PC are closer to one compared to other model selection criteria. The estimated values of precision, sensitivity, and MCC changed also but the “ranking” of the model selection criteria was practically identical to the one presented in Tables 1 and 2. See the Supplementary materials for graphical illustration of these additional simulation results.

Finally, we tested how the gap-com statistic depends on the choice of the community detection algorithm. We generated data from the star, cluster, and the Barabási–Albert model and computed the gap-com statistic using the Walktrap, the Fast and Greedy (Clauset et al. 2004), and the Propagating labels (Raghavan et al. 2007) community detection algorithms. Overall, although the gap-com statistic and the corresponding tuning parameter value are different when the community detection algorithm is changed, there is no striking difference between the averaged NMI values between these community detection algorithms. The detailed results are shown in the Supplementary Materials.

For graphical illustration of all simulation results (e.g. more detailed dispersion metrics), see the Supplementary Materials.

### *Staphylococcus aureus* DREAM5 data results

**Fig. 4.**
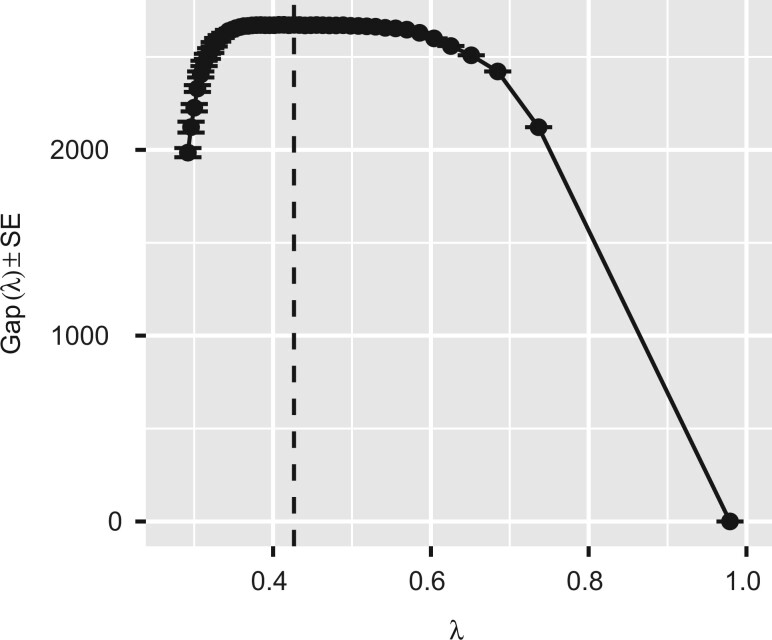
The gap-com statistic used to determine the optimal value of the tuning parameter for sparse network estimate of the DREAM5 *S. aureus* network (the vertical dashed line). The gap-com statistic is maximized with a tuning parameter value resulting in 137 clusters in the network. From these indentified clusters 4 contain more than single nodes. The standard errors are illustrated with horizontal lines around the corresponding values of Gap(λ).

There are 137 clusters in the sparse co-expression network when the gap-com statistic is used as the model selection criterion (see also Fig. 4). Note that Fig. 4 gives the impression that there are numerous identical gap-com statistic values which are also maximal. However, there are only 3 identical values which maximize Gap(λ), although this might not be clearly visible in Fig. 4. We choose the co-expression network model with the smallest sparsity level following the parsimony principle. As noted by Tibshirani et al. (2001) it is important to examine the whole gap curve in case there is cluster structure which does not maximize the gap statistic but is visible as multiple local maxima on the curve. In this particular case a wider range of tuning parameter values (roughly in the interval [0.37,0.60]) might also represent a group of competing network models. Thus, choosing the co-expression network model corresponding, e.g. to the tuning parameter value 0.6 could arguably be justified in this case.

There are 133 isolated nodes in the network selected with the gap-com statistic. The Walktrap identified 688 clusters (513 isolated nodes) from the co-expression network selected with PC and 129 clusters (114 isolated nodes) from the network selected with AGNES. None of the criteria selected the same sparse co-expression network model. The sparsity-levels of different network estimates were as follows: gap-com, 13.469%; StARS, 0.000%; PC, 0.612%; and AGNES, 30.000%. The total number of possible edges in the graph is 3,946,645. StARS selected a drastically sparse graph with only one edge. One can change this by either (1) increasing the extra tuning parameter of the StARS procedure even further (relaxing the sparsity assumption) or (2) using some other method to estimate the parameters of the co-expression network model. Nevertheless, we leave the results as they are to illustrate the difference between the model selection criteria we have used.

Co-expression nodes corresponding to the pathogen module in the network models selected using the gap-com statistic and AGNES share similar characteristics in the gap-com graphs and the AGNES graphs. The pathogen module constructed from the graph selected with PC was very sparse and the number of edges of PC Pathogen module is 19. Nodes (genes) of these Pathogen modules share many similarities with the community network shown in Marbach et al. (2012). For example, 10 neighbors out of the 11 neighbors of SAV2357 (transcription regulator) in the gap-com graph and 14 genes out of the 20 neighbors in the AGNES graph are also neighbors of SAV2357 in the Pathogen module. In addition, 9 out of 10 of the neighbors of SAV2553 (tetracycline repressor) in the gap-com graph are also neighbors of SAV2553 in the Pathogen module and they cover the whole neighborhood of the community network Pathogen module (with one false positive). Moreover, the Walktrap algorithm places the neighborhood of SAV2553 in the same community. The high-quality figures of the pathogenic module selected using the gap-com statistic, StARS, PC, and AGNES are found in the Supplementary Materials.

None of the methods identified exactly the same set of hub genes. The SAV0996 gene is the top hub gene in the networks selected using the gap-com statistic and the second highest hub node in the network selected using AGNES. The list of the neighborhood of the SAV0996 gene detected in the gap-com network are found in the Supplementary Materials. There are no decoy genes among the top 100 hub genes in the co-expression network estimates selected with the gap-com statistic, PC, and AGNES.

The pre-processing of the data matters in this analysis but without deeper knowledge about the data we cannot examine how this affects different network construction methods [see Supplementary Note 10.3 of Marbach et al. (2012)].

### Discussion

We have proposed a novel gap statistic in a new context that allows the detection of the upper-level hierarchy structure of the sparse network model (the number of network communities). Using the random graph model to estimate the expected number of clusters under the E-R model is more time efficient compared with the data permutation strategy and the graphical models selected with either of these strategies are practically the same. In addition, parallel computing can considerably reduce the running time of the gap-com procedure.

Finding gene clusters and hub genes from a complex gene co-expression network are demanding problems. Moreover, there is no mathematically unified definition for a network community (see Fortunato 2010, pp. 83–84) which leaves the detection of representative clusters open to interpretation. Like the original gap statistic, the gap-com statistic depends on the method used to construct the undirected graphical model and on the community detection algorithm. This results in numerous combinations of estimation procedures and community detection algorithms which have direct influence on the final graphical model selected with the gap-com statistic.

In this study, we have concentrated on undirected complex network models with a clear cluster structure. It would be interesting to try how the gap-com statistic works if one considers overlapping or hierarchical communities in the community detection algorithm. As far as we know, no other graphical model selection criterion can take overlapping communities into account. This would be an interesting direction for future studies, considering that our simulations with the Barabási–Albert model illustrate that the gap-com statistic returns a sensible network model although the unambiguous cluster structure is not considered.

While not discussed in this study, the gap-com statistic is readily applicable to weighted graphs and for discrete data. It would be straightforward to apply the gap-com statistic in a setting where the overall structure and sparsity of the complex network is controlled with multiple hyperparameters. For example, in the work of Liu and Ihler (2011), the authors propose an additional power law regularization to estimate non-zero entries of sparse precision matrices that can be used to construct a gene co-expression network with a hub structure. One could use the gap-com statistic to select the regularization coefficients of this approach.

## Data availability

A demo script and R codes for reproducing all the analyses and figures represented in this study are available at GitHub under the GPL license, https://github.com/markkukuismin/gap-com. The authors affirm that all data necessary for confirming the conclusions of the article are present within the article, figures, and tables.

Supplemental material is available at *G3* online.

## Supplementary Material

jkab437_Supplementary_File_S1

jkab437_Supplementary_Data
