# Supplementary material for: Gap-com: general model selection criterion for sparse undirected gene networks with nontrivial community structure
Source: G3 (Bethesda). 2021 Dec 21;12(2):jkab437. doi: 10.1093/g3journal/jkab437 (PMC9210289; doi:10.1093/g3journal/jkab437)
Supplement: jkab437_Supplementary_Data [file jkab437_supplementary_data.pdf]

Gap-com: General model selection criterion for sparse undirected gene networks with non-trivial community structure - Supplementary materials

Markku Kuismin[1,2,4], Fatemeh Dodangeh[1], and Mikko J. Sillanpää*[1,2,3]

[1]*Research Unit of Mathematical Sciences, University of Oulu, Finland*
[2]*Biocenter Oulu, University of Oulu, Finland*
[3]*Infotech Oulu, University of Oulu, Finland*
[4]*School of Computing, University of Eastern Finland, Finland*

September 22, 2021

1 Simulation results

Box plots of the simulation results reported in Table 1 in the main document using hard thresholding or BigQuic (Hsieh et al., 2013) are reported in subsections 1.1 and 1.5.

To compare the simulation results with the "ground truth" graphs, we used the Walktrap community detection algorithm to determine the true number of clusters in the Barabási–Albert (scale-free) and E-R graph models. The results were as follows: the Walktrap algorithm detected 40 clusters

*To whom correspondence should be addressed: mikko.sillanpaa@oulu.fi

in the simulated Barabási–Albert (scale-free) graph model, and 5 in the simulated E-R graph model. Both the cluster graph model and the **star graph** model include ten disjoint clusters.

1.1 Hard thresholding, binary classification results

Figures in this subsection summarize the sensitivity (Sen), the precision (Pre), and the Matthews correlation coefficient (MCC) of the simulation runs where different model selection criteria are compared with each other. In addition, we used the normalized mutual information (NMI) measure to evaluate how close the detected communities are with the ground truth clusters.

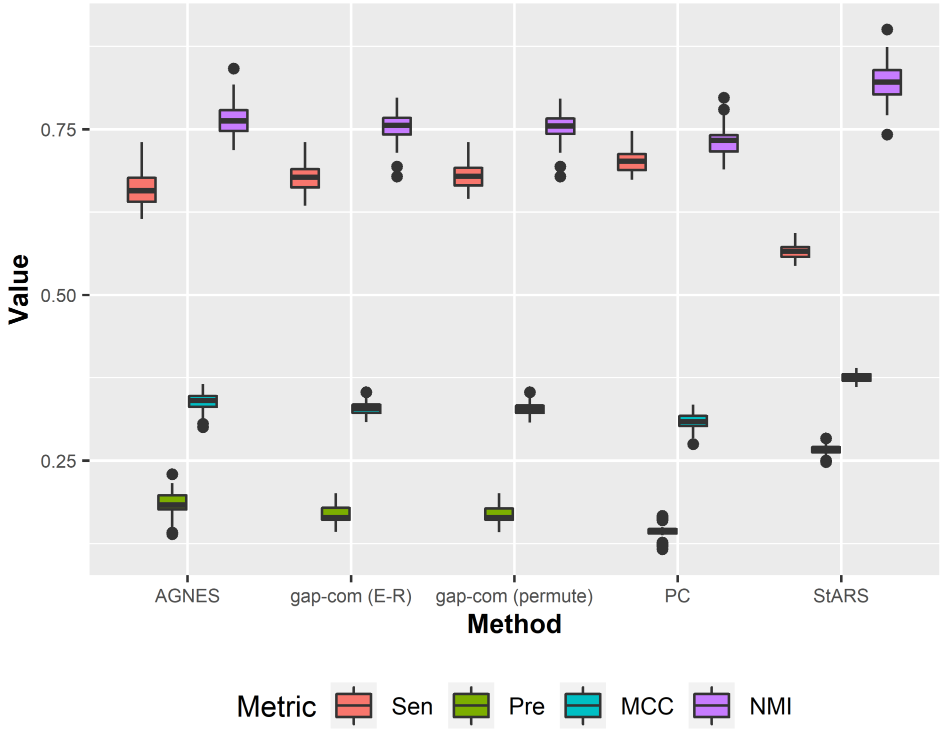

Figure 1: Hard thresholding, the **cluster graph** model, $p = 500$, $n = 200$.

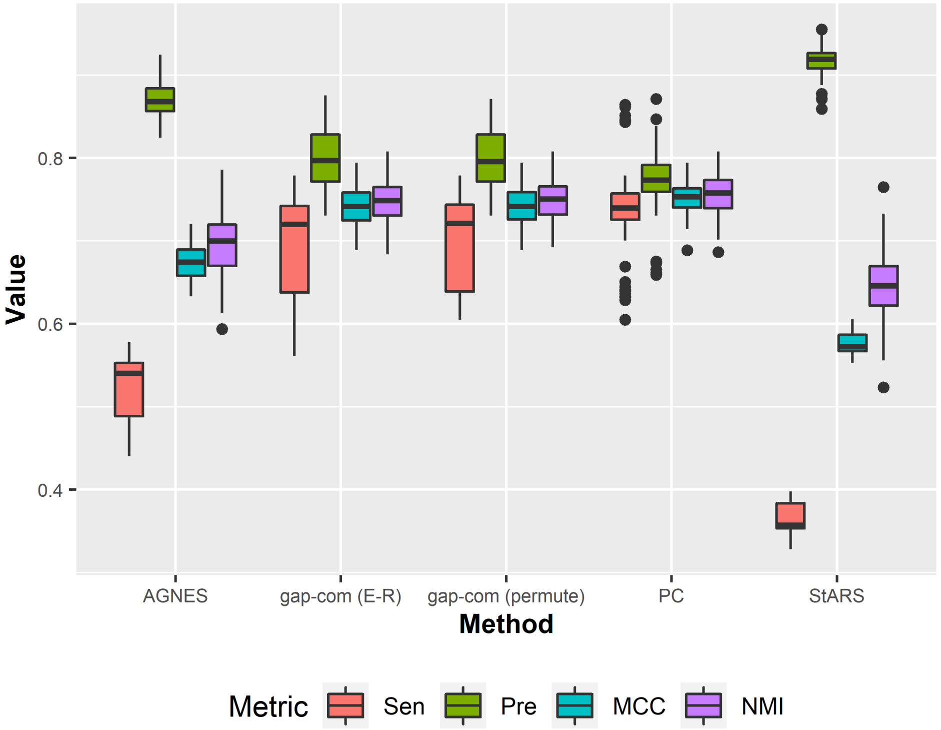

Figure 2: Hard thresholding, the **star graph** model, $p = 500$, $n = 200$.

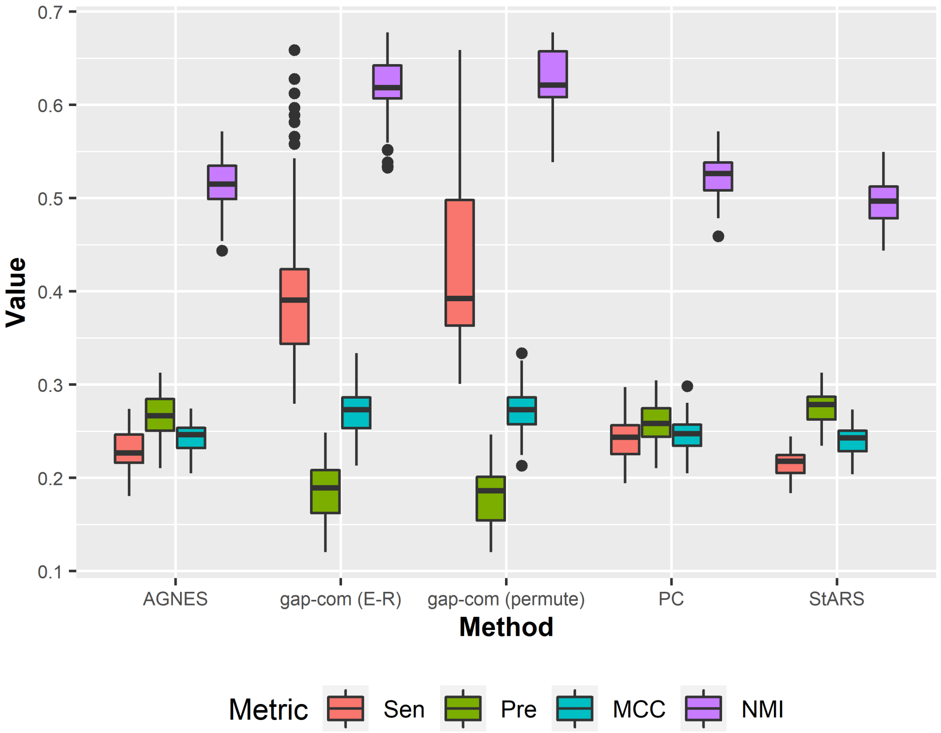

Figure 3: Hard thresholding, the **Barabási–Albert (scale-free) graph** model, $p = 500$, $n = 200$.

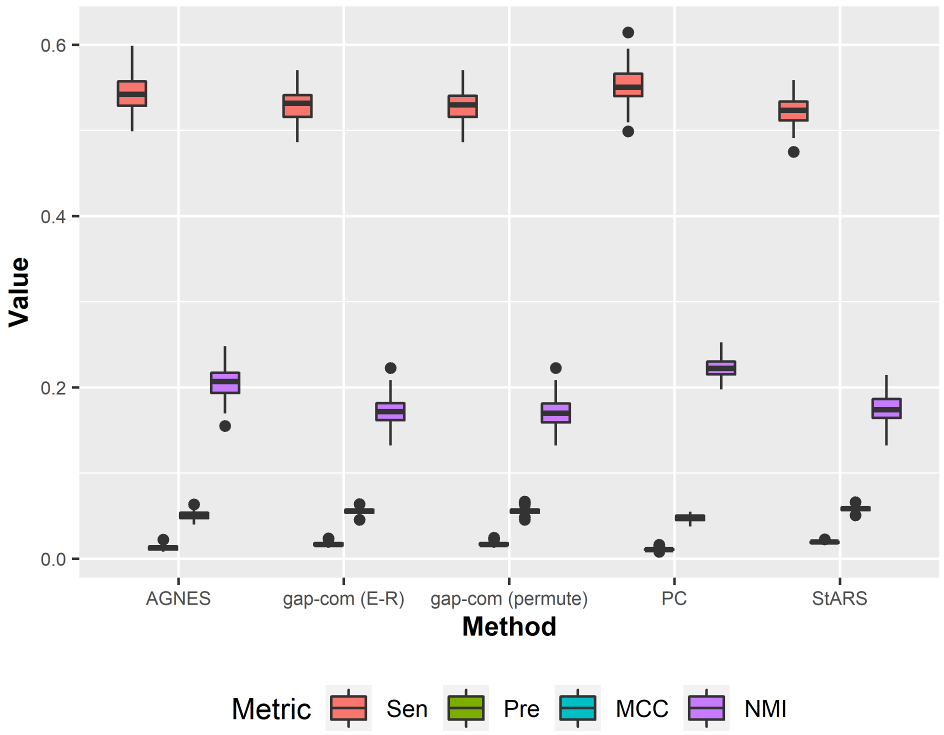

Figure 4: Hard thresholding, the **Erdos-Renyi (random) graph** model, $p = 500$, $n = 200$.

1.2 Hard thresholding, the number of detected clusters

In each scenario we used the Walktrap algorithm to detect the communities in the estimated graph. In the Walktrap algorithm of the R package `igraph` we set the length of the random walks to 4 and calculate the membership vector for the split corresponding to the highest modularity value. The results are summarized in Figures below.

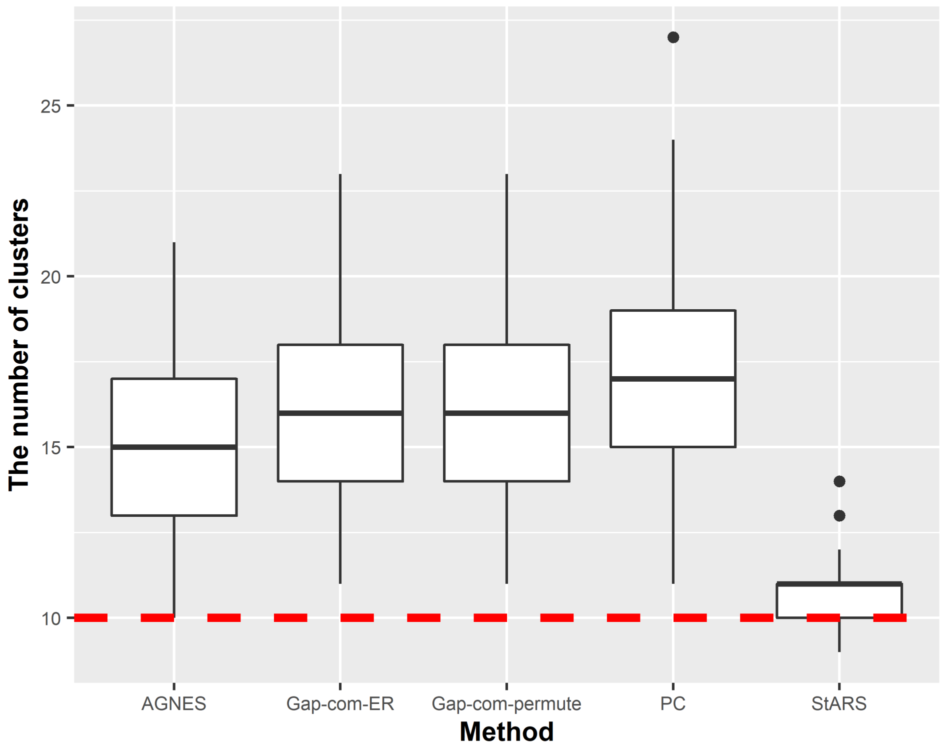

Figure 5: The number of clusters detected from the **cluster graph** while the hard thresholding is used in the graphical model construction and $p = 500$, $n = 200$. The dashed horizontal line illustrates the true number of clusters.

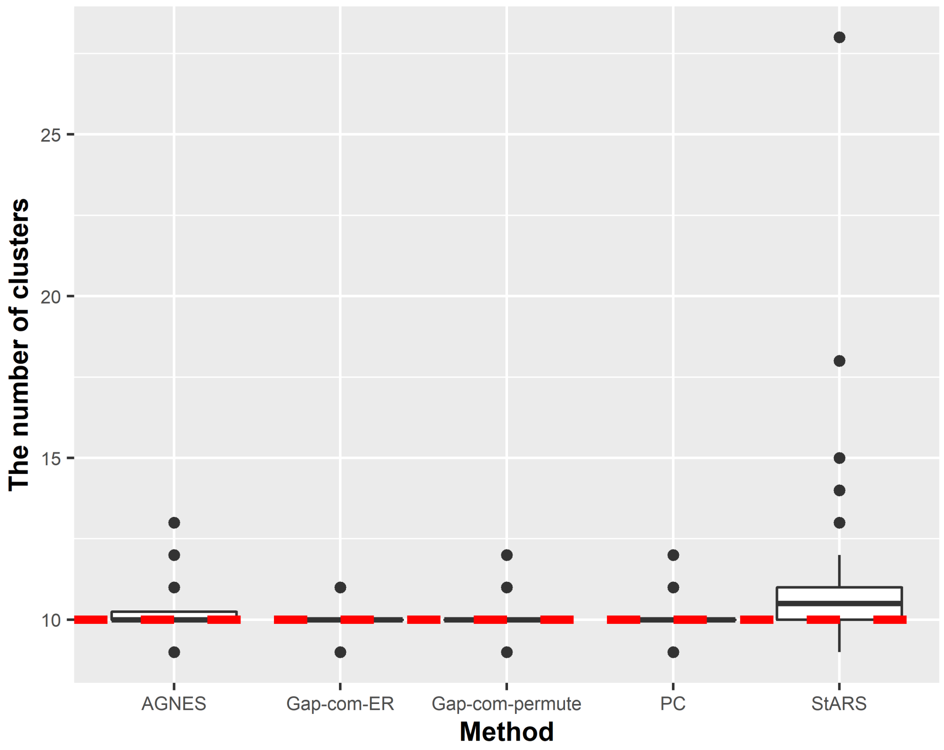

Figure 6: The number of clusters detected from the **star graph** while the hard thresholding is used in the graphical model construction and $p = 500$, $n = 200$. The dashed horizontal line illustrates the true number of clusters.

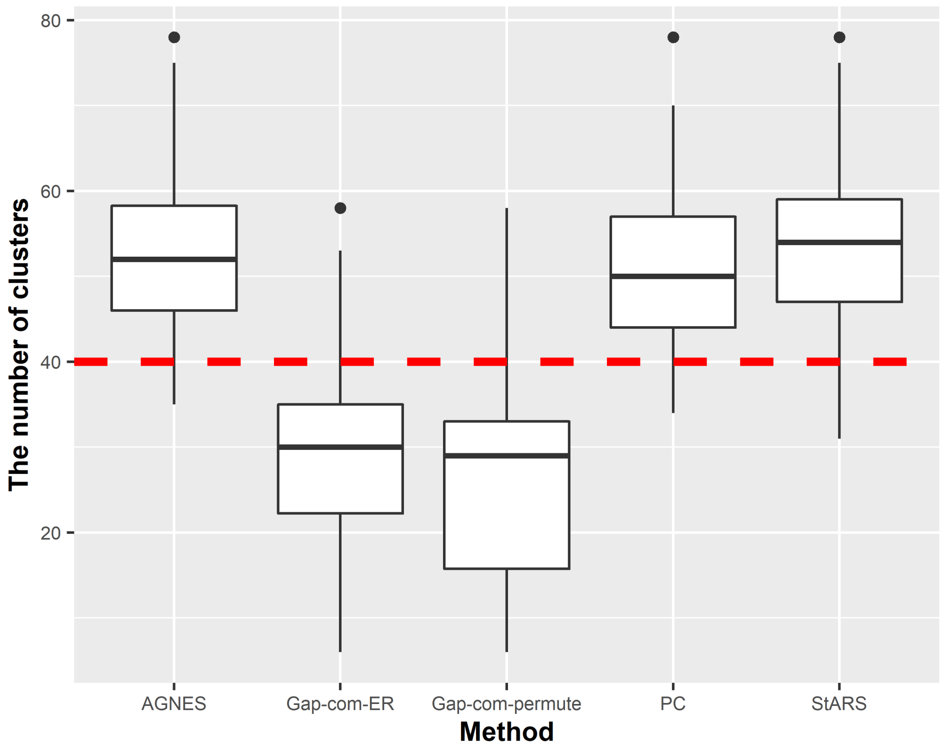

Figure 7: The number of clusters detected from the **Barabási–Albert (scale-free) graph** while the hard thresholding is used in the graphical model construction and $p = 500$, $n = 200$. The dashed horizontal line illustrates the true number of clusters (40).

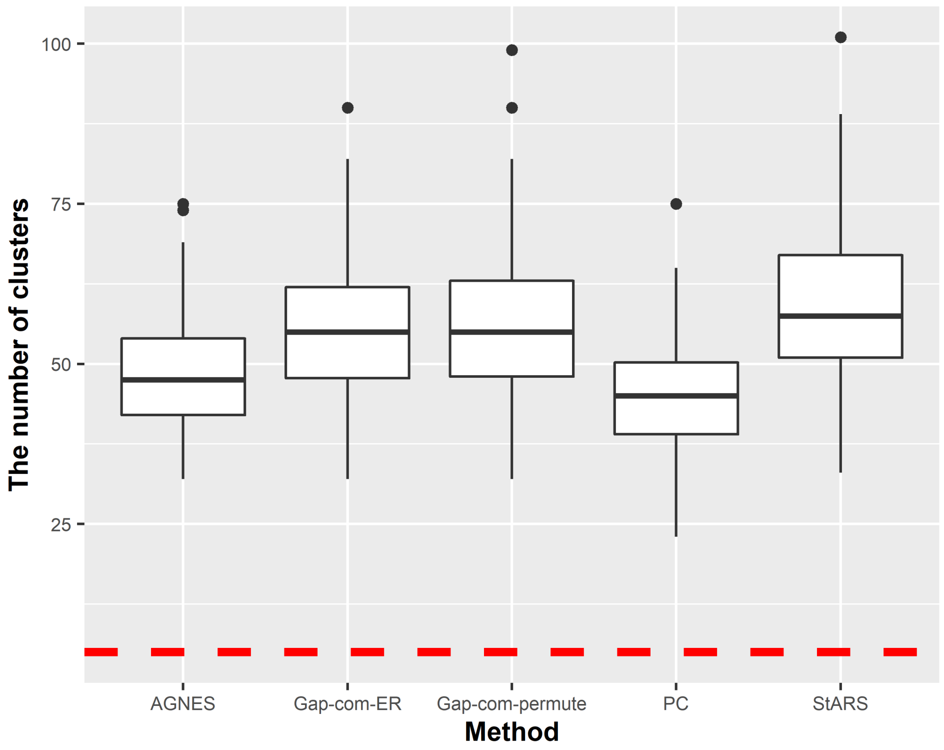

Figure 8: The number of clusters detected from the Erdos-Renyi (random graph) while the hard thresholding is used in the graphical model construction and $p = 500$, $n = 200$. The dashed horizontal line illustrates the true number of clusters (5).

1.3 Hard thresholding, graph modularity

We calculated the modularity of the selected graphical model with respect to the given community structure detected with the Walktrap community detection algorithm. The results are summarized in Figures below.

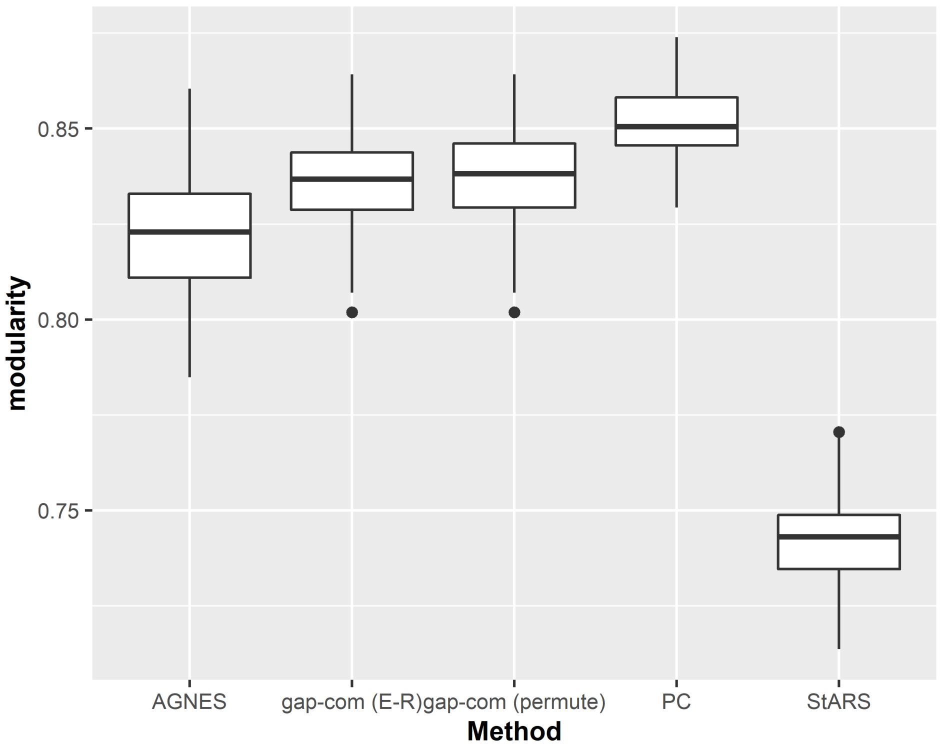

Figure 9: Modularity averaged over 100 simulation runs when hard thresholding is used to detect the sparsity pattern of the correlation matrix. The ground truth graph follows the **cluster graph** model, $p = 500$, $n = 200$.

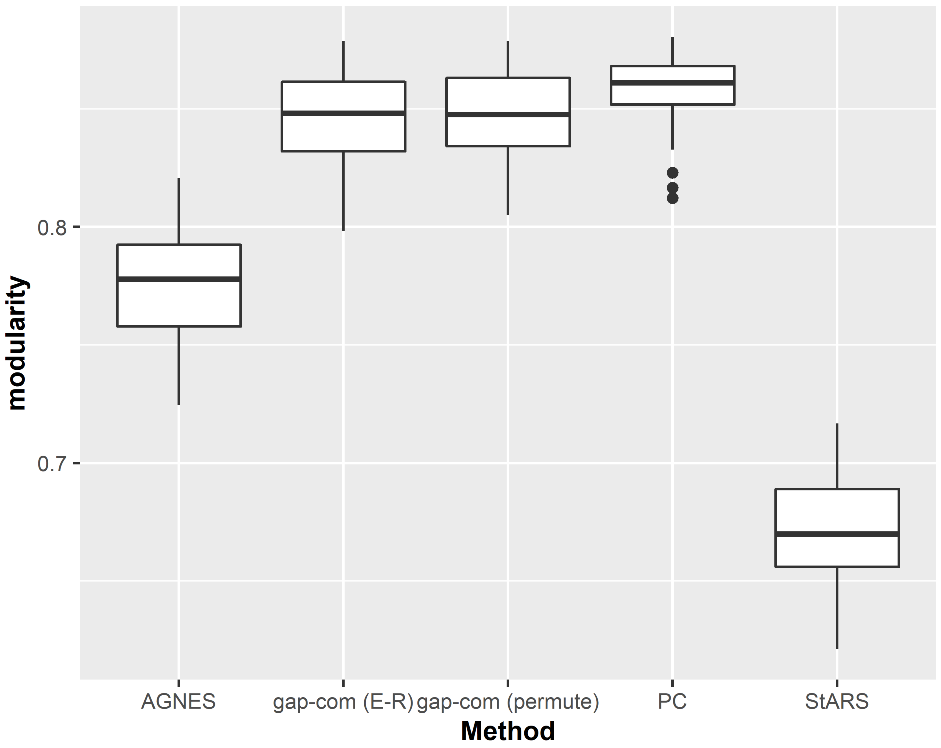

Figure 10: Modularity averaged over 100 simulation runs when hard thresholding is used to detect the sparsity pattern of the correlation matrix. The ground truth graph follows the **star graph** model, $p = 500$, $n = 200$.

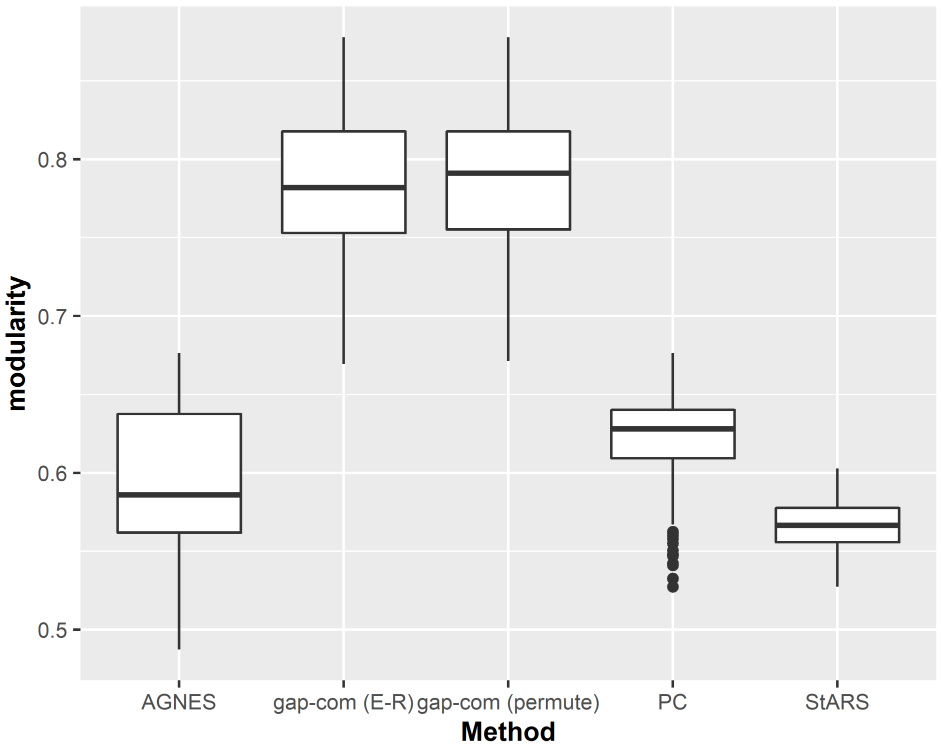

Figure 11: Modularity averaged over 100 simulation runs when hard thresholding is used to detect the sparsity pattern of the correlation matrix. The ground truth graph follows the **Barabási–Albert (scale-free) graph** model, $p = 500$, $n = 200$.

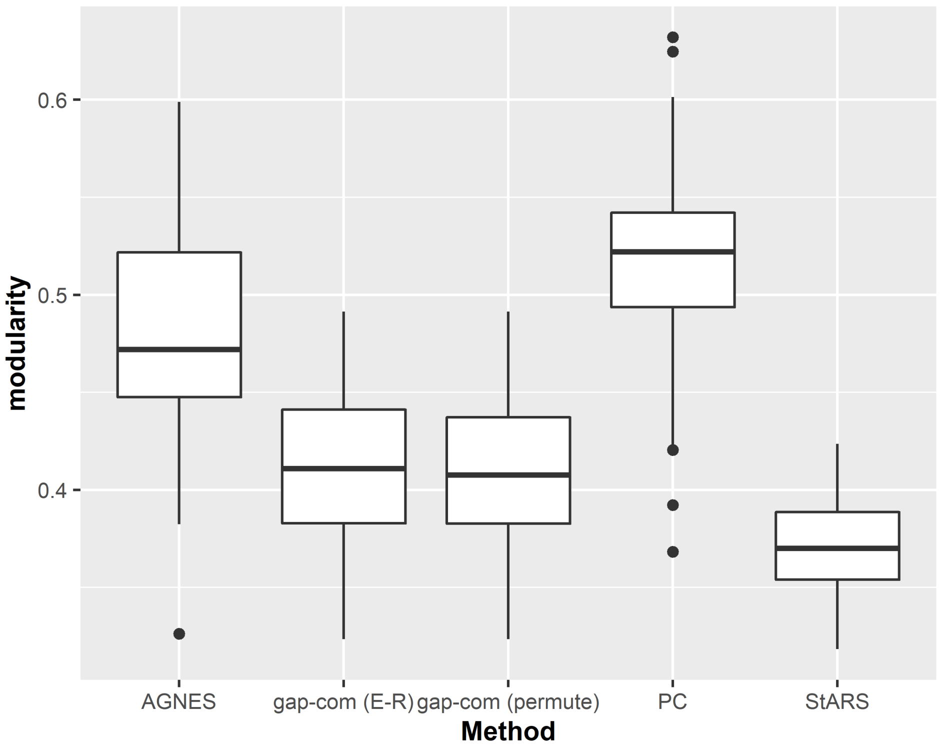

Figure 12: Modularity averaged over 100 simulation runs when hard thresholding is used to detect the sparsity pattern of the correlation matrix. The ground truth graph follows the **Erdos-Renyi (random) graph** model, $p = 500$, $n = 200$.

1.4 Hard thresholding, selected tuning parameters

All tuning parameter values selected during the simulation runs when the hard thresholding of the absolute values of the Pearson correlation coefficient is used to estimate the sparsity pattern of the correlation matrix are summarized in the boxplots below.

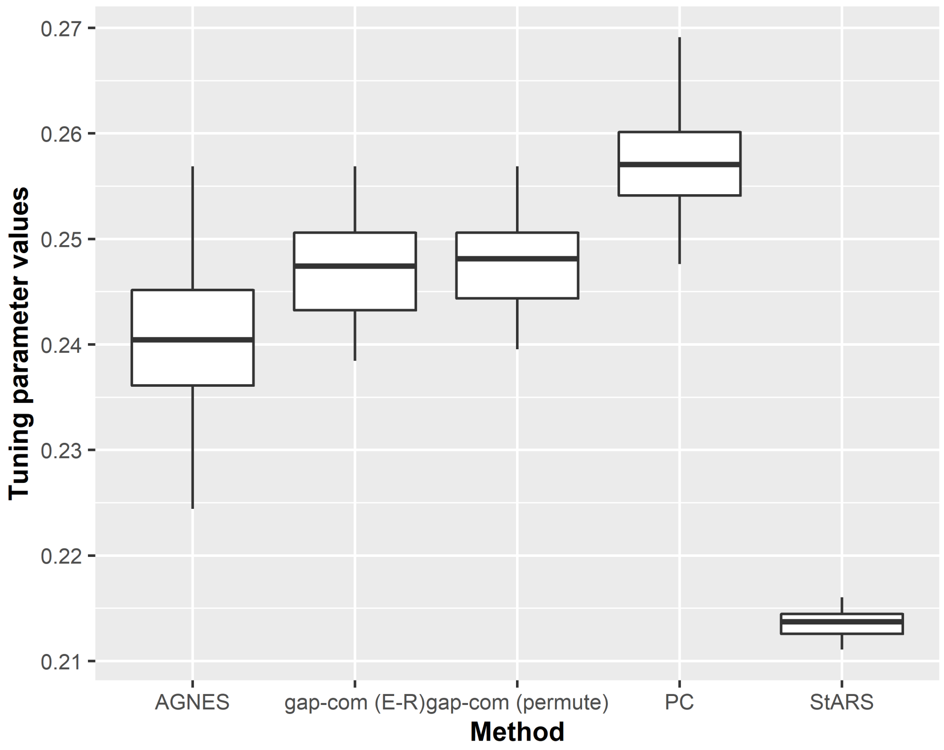

Figure 13: Selected tuning parameter values while the graph is estimated using the hard thresholding. The data depends on the structure of the **cluster graph** model, $p = 500$, $n = 200$.

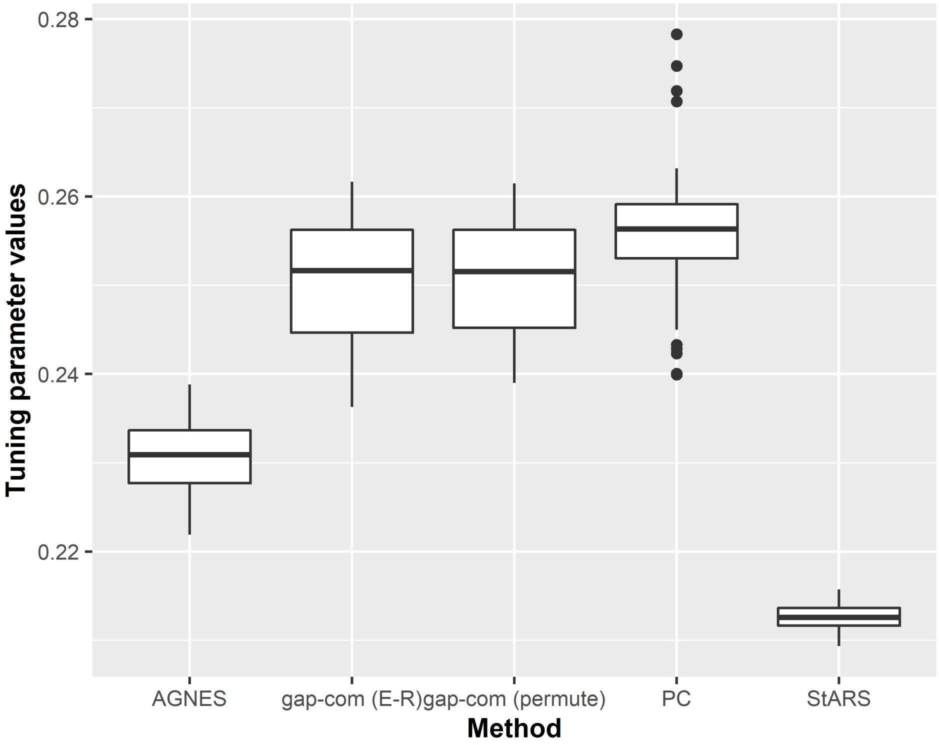

Figure 14: Selected tuning parameter values while the graph is estimated using the hard thresholding. The data depends on the structure of the **star graph** model, $p = 500$, $n = 200$.

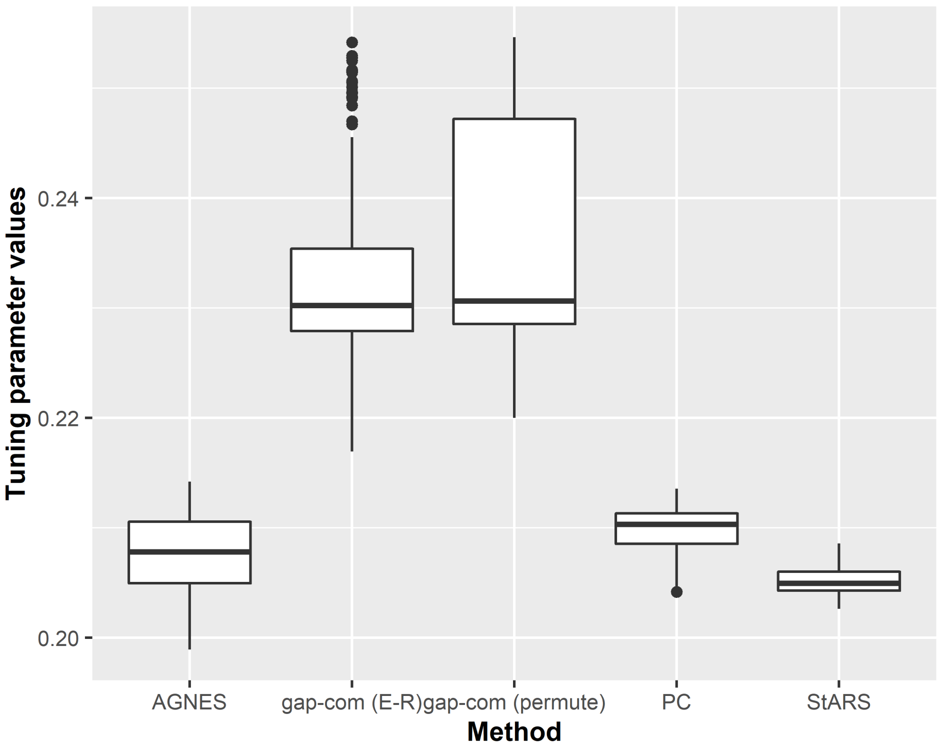

Figure 15: Selected tuning parameter values while the graph is estimated using the hard thresholding. The data depends on the structure of the **Barabási–Albert (scale-free) graph** model, $p = 500$, $n = 200$.

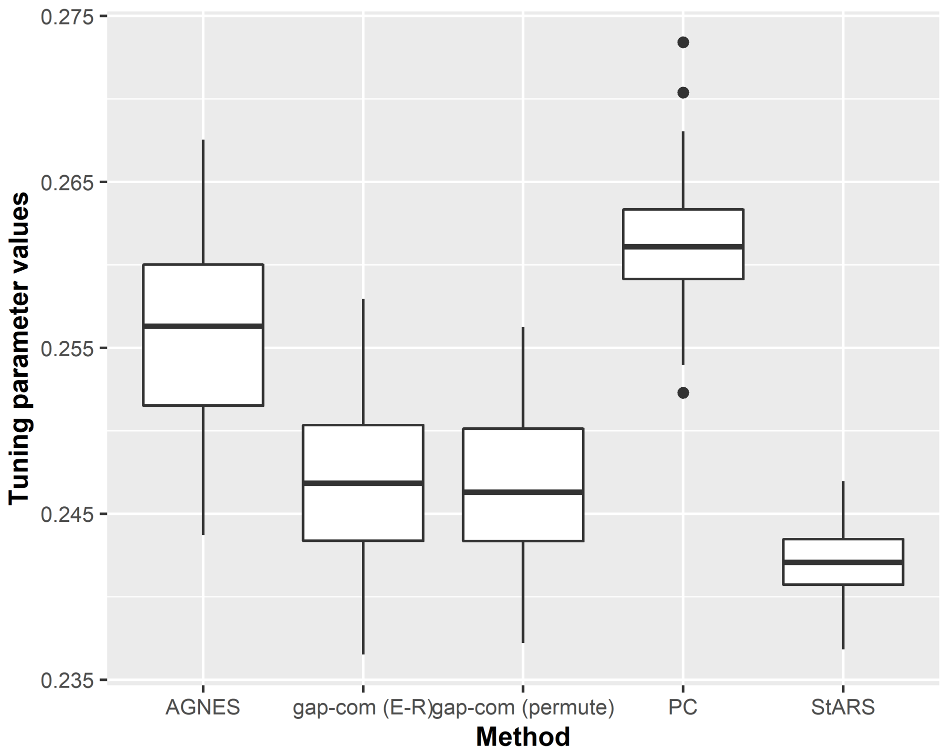

Figure 16: Selected tuning parameter values while the graph is estimated using the hard thresholding. The data depends on the structure of the **Erdos-Renyi (random) graph** model, $p = 500$, $n = 200$.

1.5 BigQuic method, binary classification results

Figures in this subsection summarize the sensitivity (Sen), the precision (Pre), and the Matthews correlation coefficient (MCC) of the simulation runs where different model selection criteria are compared with each other. In addition, we used the normalized mutual information (NMI) measure to evaluate how close the detected communities are with the ground truth clusters.

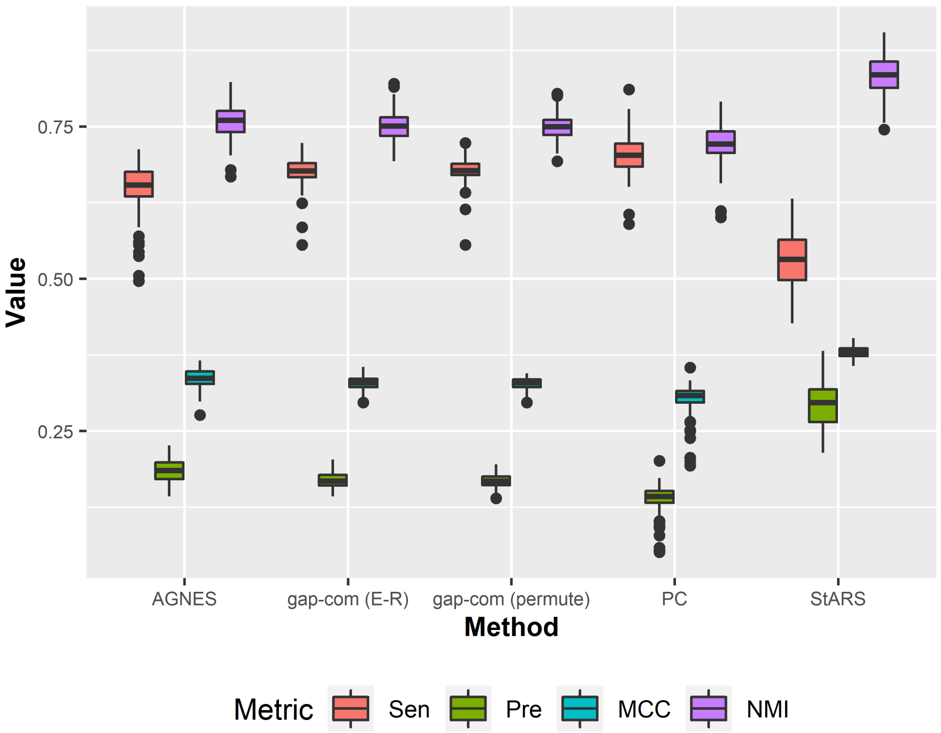

Figure 17: BigQuic, the **cluster graph** model, $p = 500$, $n = 200$.

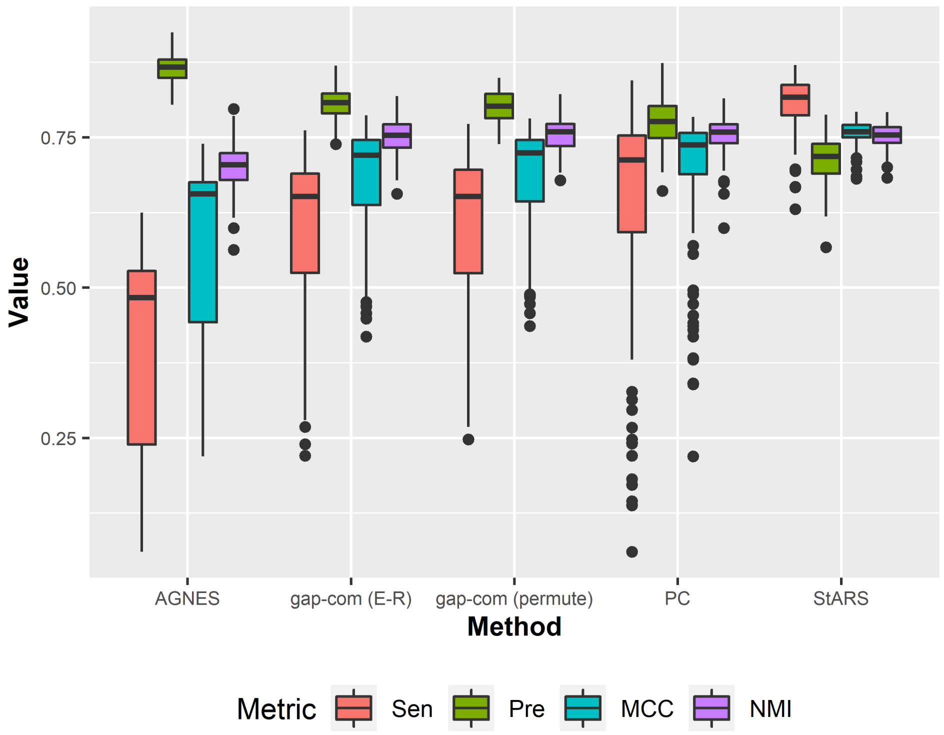

Figure 18: BiqQuic, the **star graph** model, $p = 500$, $n = 200$.

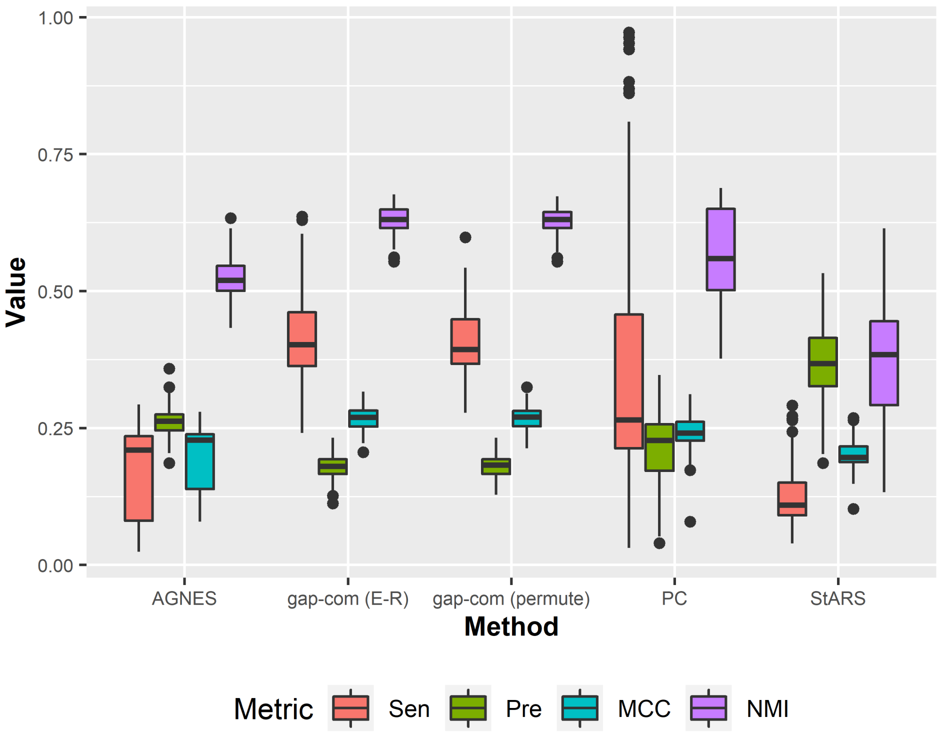

Figure 19: BigQuic, the **Barabási–Albert (scale-free) graph** model, $p = 500$, $n = 200$.

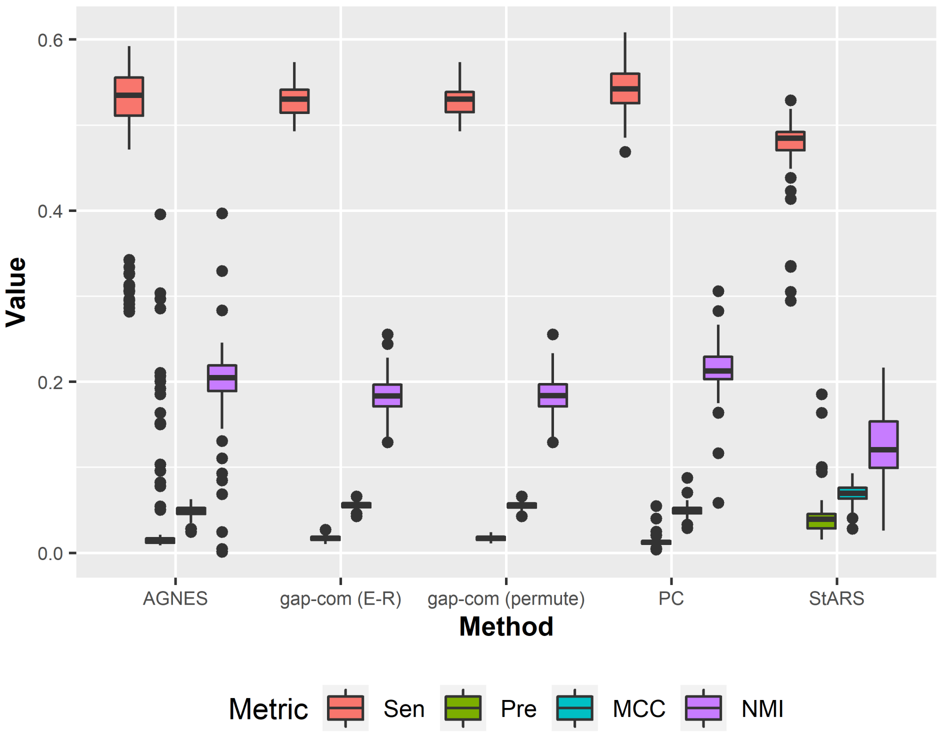

Figure 20: BigQuic, the **Erdos-Renyi (random) graph** model, $p = 500$, $n = 200$.

1.6 BigQuic, the number of detected clusters

In each scenario we used the Walktrap algorithm to detect the communities. In the Walktrap algorithm of the R package `igraph` we set the length of the random walks to 4 and calculate the membership vector for the split corresponding to the highest modularity value. The results are summarized in Figures below.

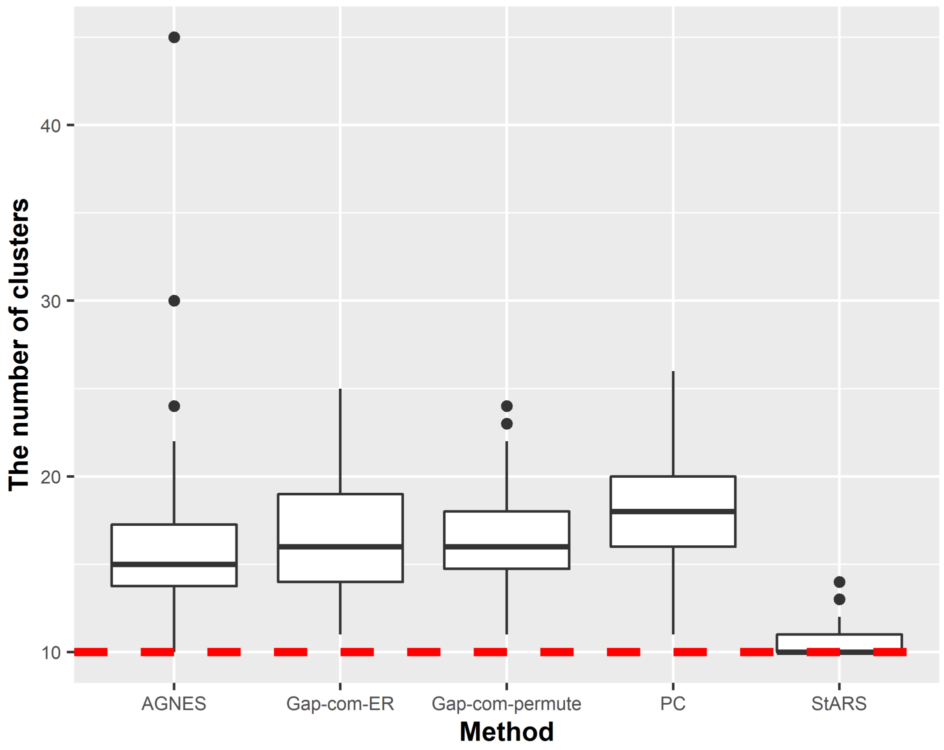

Figure 21: The number of clusters detected from the **cluster graph** while the BigQuic is used in the graphical model construction and $p = 500$, $n = 200$. The dashed horizontal line illustrates the true number of clusters.

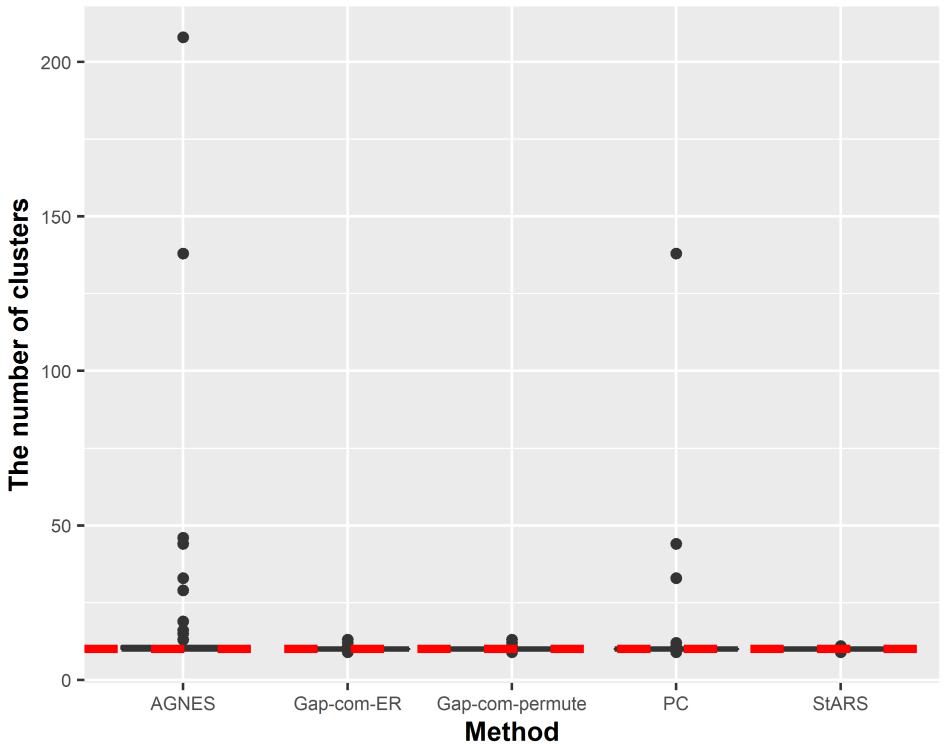

Figure 22: The number of clusters detected from the **star graph** while the BigQuic is used in the graphical model construction and $p = 500$, $n = 200$. The dashed horizontal line illustrates the true number of clusters.

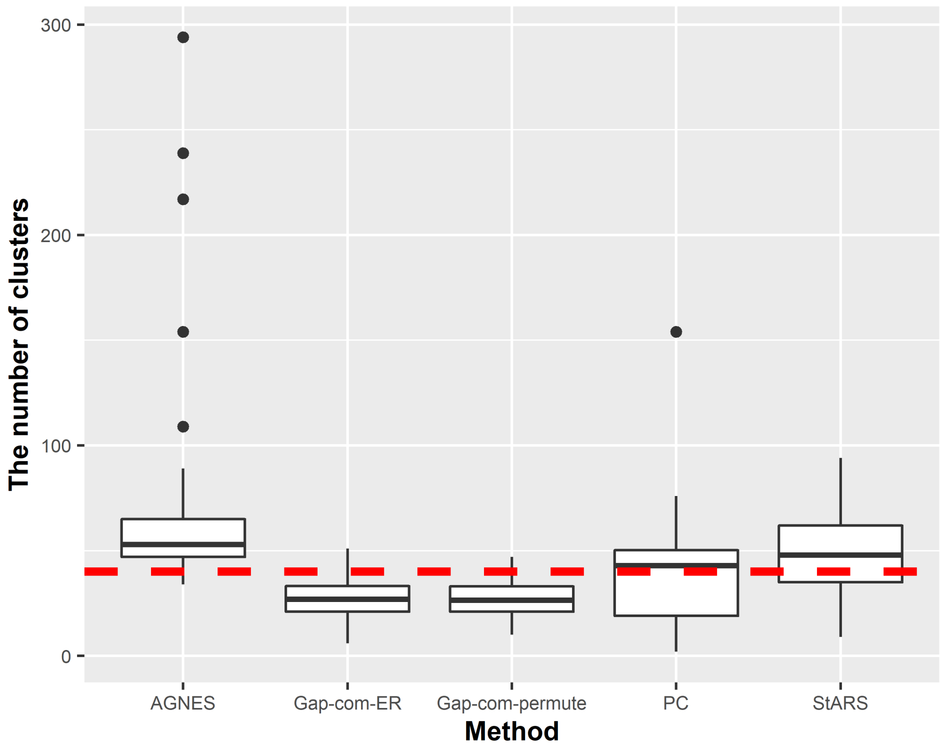

Figure 23: The number of clusters detected from the **Barabási–Albert (scale-free) graph** while the BigQuic is used in the graphical model construction and $p = 500$, $n = 200$. The dashed horizontal line illustrates the true number of clusters (40).

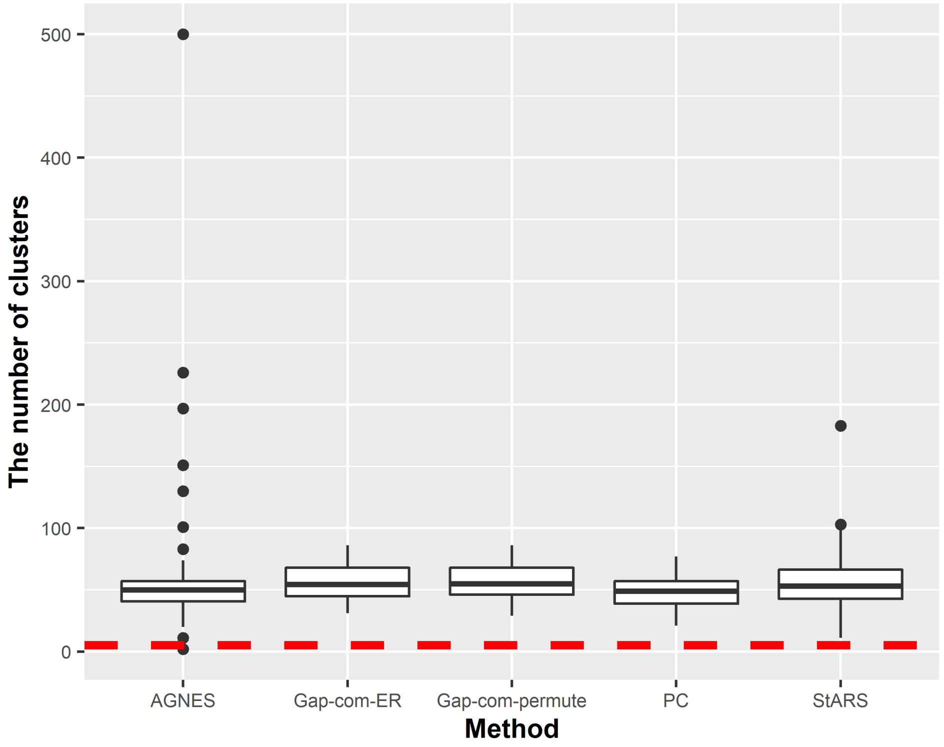

Figure 24: The number of clusters detected from the Erdos-Renyi (random graph) while the BigQuic is used in the graphical model construction and $p = 500$, $n = 200$. The dashed horizontal line illustrates the true number of clusters (5).

1.7 BigQuic, graph modularity

Such as with the hard threshold method, we calculated the modularity of the selected graphical model with respect to the given community structure detected with the Walktrap community detection algorithm. The results are summarized in Figures below.

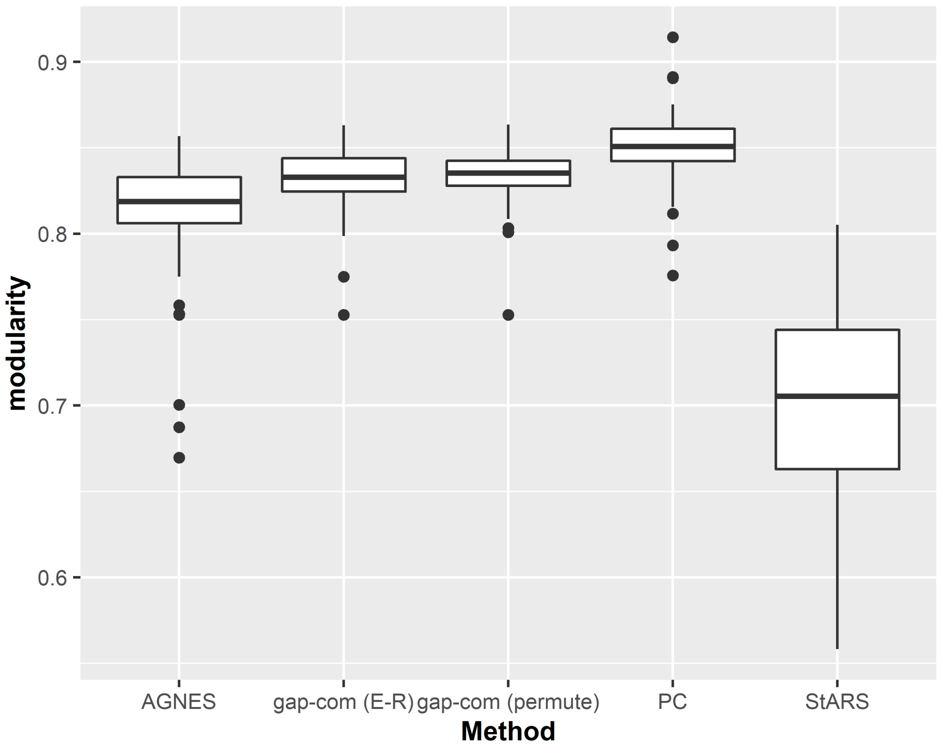

Figure 25: Modularity averaged over 100 simulation runs when BigQuic is used to detect the sparsity pattern of the correlation matrix. The ground truth graph follows the **cluster graph** model, $p = 500$, $n = 200$.

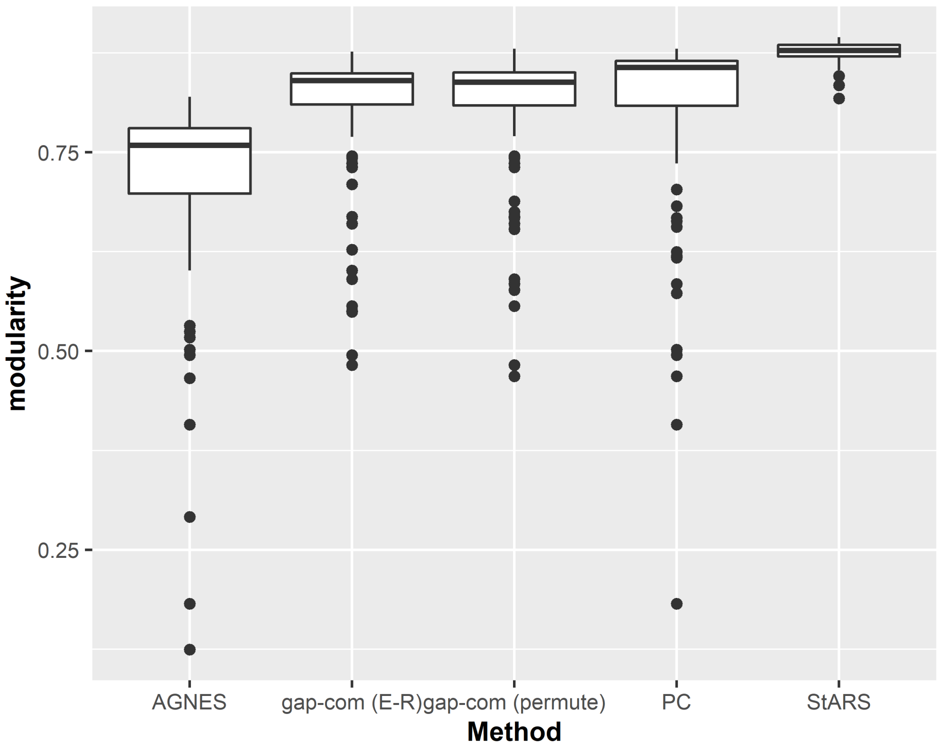

Figure 26: Modularity averaged over 100 simulation runs when BigQuic is used to detect the sparsity pattern of the correlation matrix. The ground truth graph follows the **star graph** model, $p = 500$, $n = 200$.

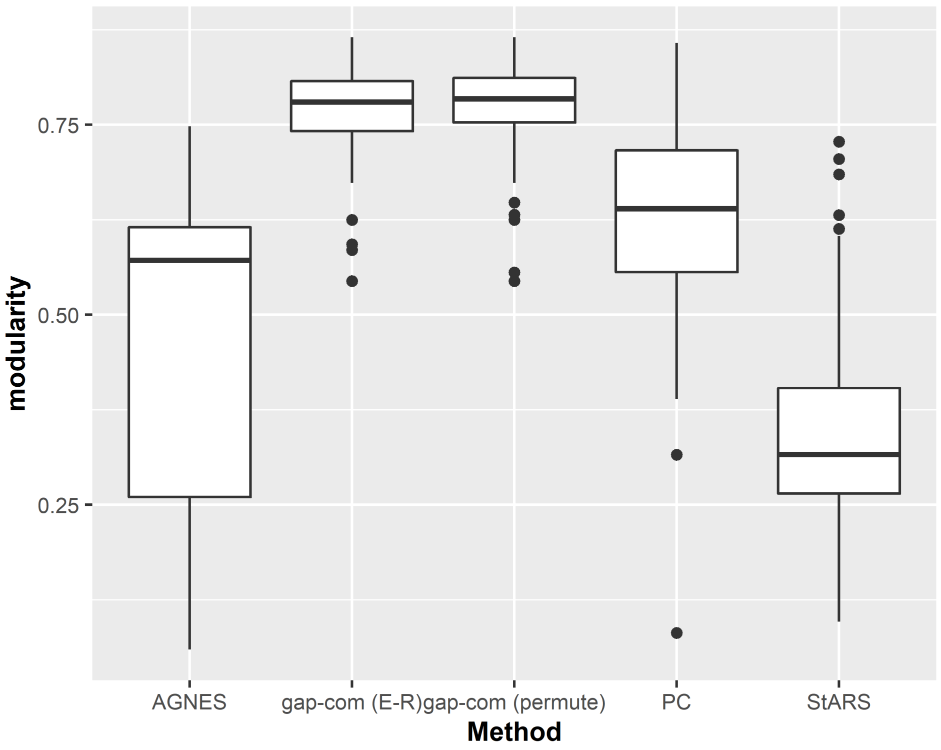

Figure 27: Modularity averaged over 100 simulation runs when BigQuic is used to detect the sparsity pattern of the correlation matrix. The ground truth graph follows the **Barabási–Albert (scale-free) graph** model, $p = 500$, $n = 200$.

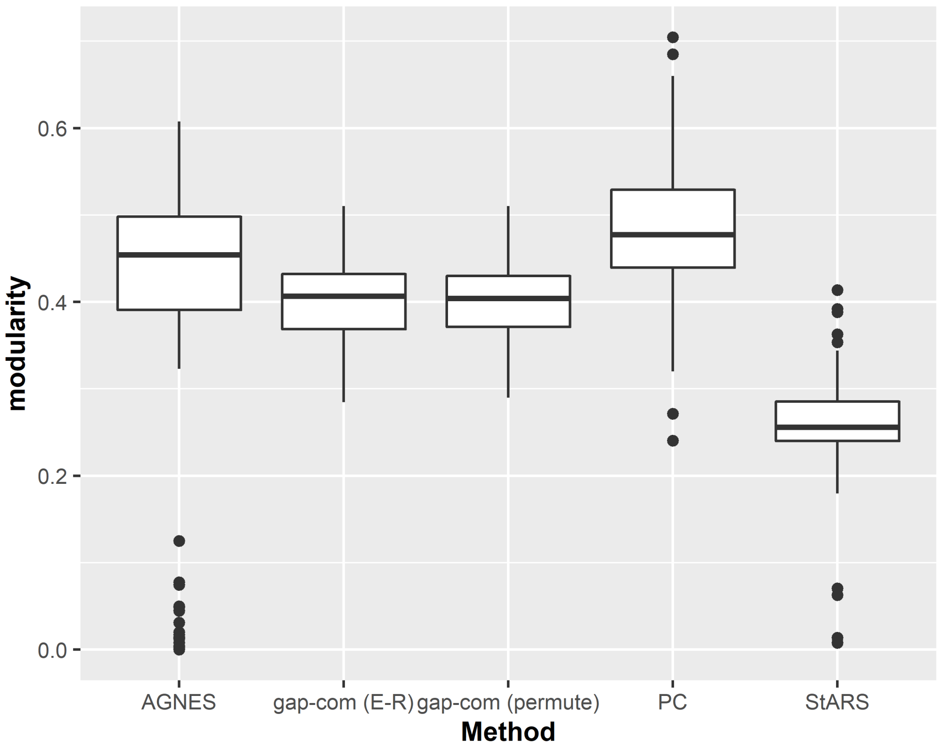

Figure 28: Modularity averaged over 100 simulation runs when BigQuic is used to detect the sparsity pattern of the correlation matrix. The ground truth graph follows the **Erdos-Renyi (random) graph** model, $p = 500$, $n = 200$.

1.8 BigQuic, selected tuning parameters

All tuning parameter values selected during the simulation runs when the BigQuic method is used to estimate the precision/partial correlation matrix are summarized in the boxplots below.

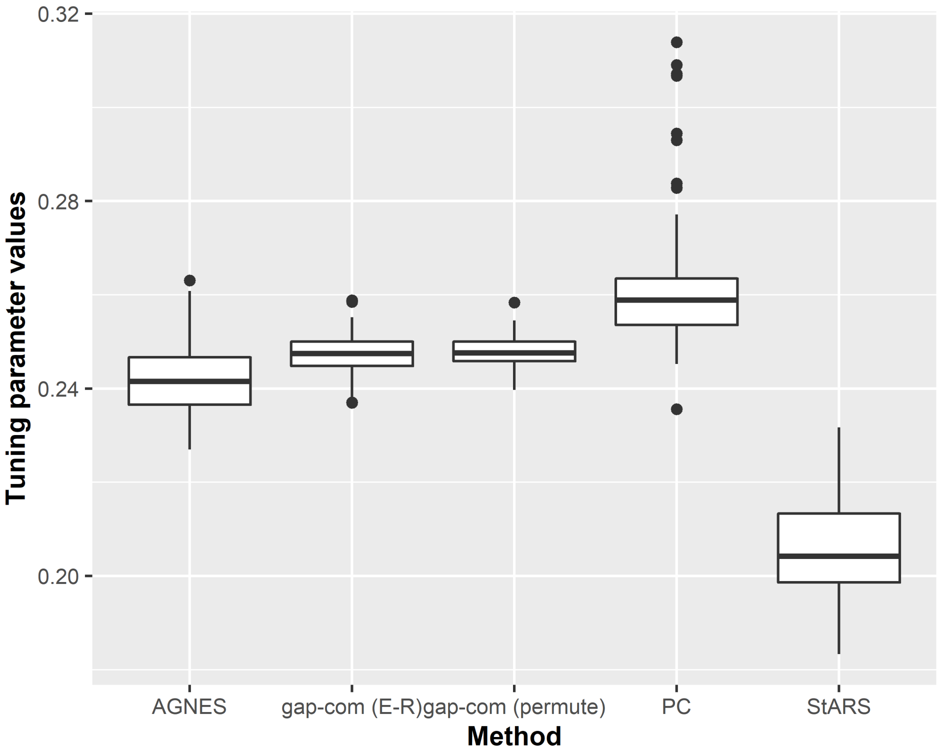

Figure 29: Selected tuning parameter values while the graph is estimated using BigQuic method. The data depends on the structure of the **cluster graph** model, $p = 500$, $n = 200$.

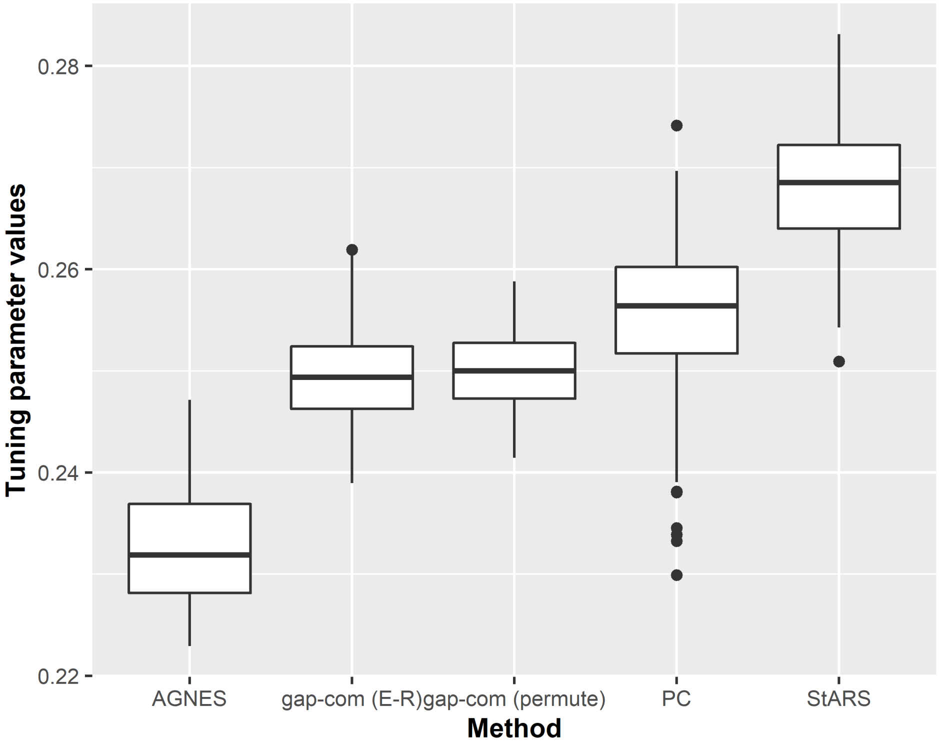

Figure 30: Selected tuning parameter values while the graph is estimated using BigQuic method. The data depends on the structure of the **star graph** model, $p = 500$, $n = 200$.

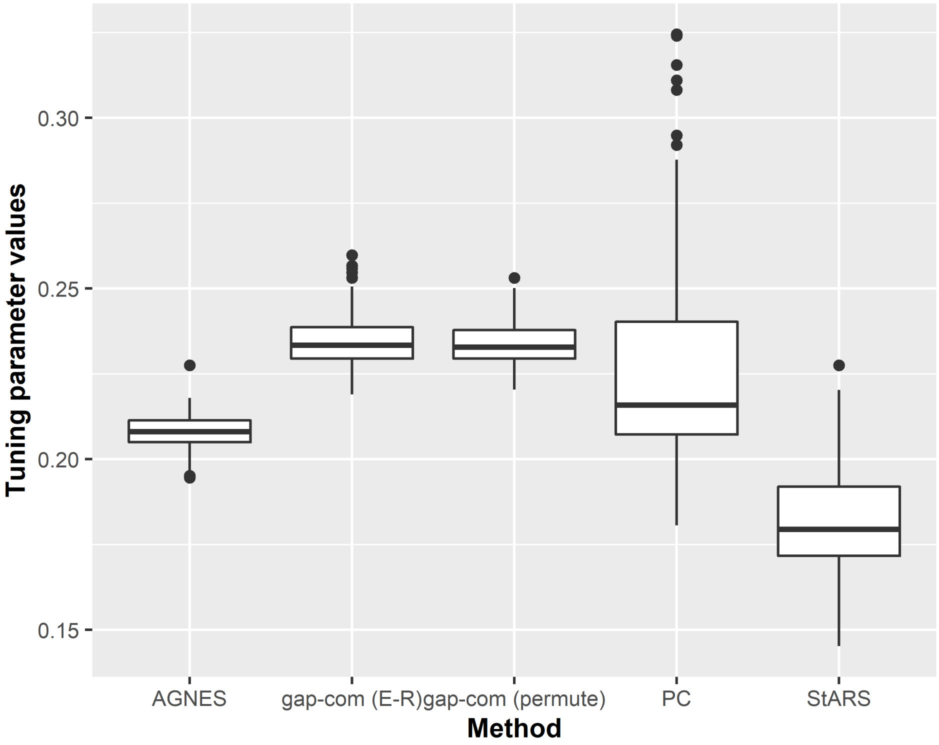

Figure 31: Selected tuning parameter values while the graph is estimated using BigQuic method. The data depends on the structure of the **Barabási–Albert (scale-free) graph** model, $p = 500$, $n = 200$.

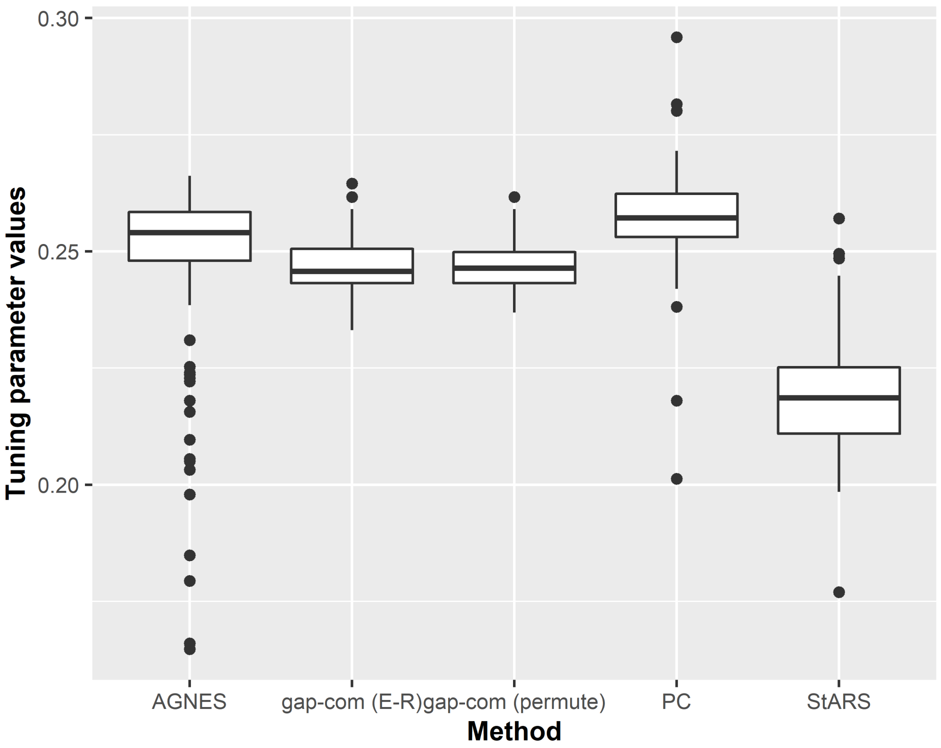

Figure 32: Selected tuning parameter values while the graph is estimated using BigQuic method. The data depends on the structure of the **Erdos-Renyi (random) graph** model, $p = 500$, $n = 200$.

2 Additional simulation results

In addition to the simulation results reported in the main paper and in the section above, we examined that,

1. how nodes with zero degree (orphan nodes) in the ground truth model affect the model selection results,
2. how does the sample size affect to the gap-com statistic,
3. how robust the gap-com statistic is to the selection of the community detection method.

Here we have not reported all statistics we reported earlier. In particular, we have reported the binary classification test results, the number of detected clusters and Normalized Mutual Information (NMI) scores. The NMI scores changes between 0 (no mutual information) and 1 (perfect correlation). We only used the hard thresholding method. Summarises of these simulation runs are reported in subsections 2.1, 2.2, and 2.3 below.

2.1 Orphan nodes

We repeated the simulations presented in the main paper but this time we removed approximately 25% of the edges at random in the ground truth graphical model to include independent random variables into the data. The averaged binary classification and NMI values are summarized in the boxplots below.

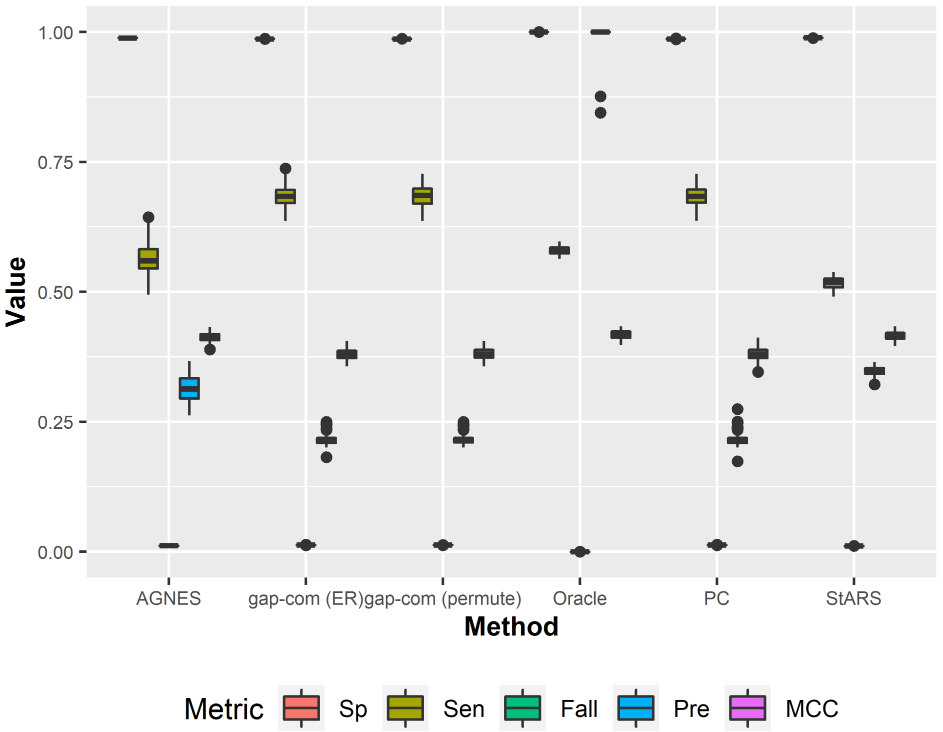

Figure 33: Hard thresholding, the **cluster graph model with orphan nodes**, $p = 500$, $n = 200$.

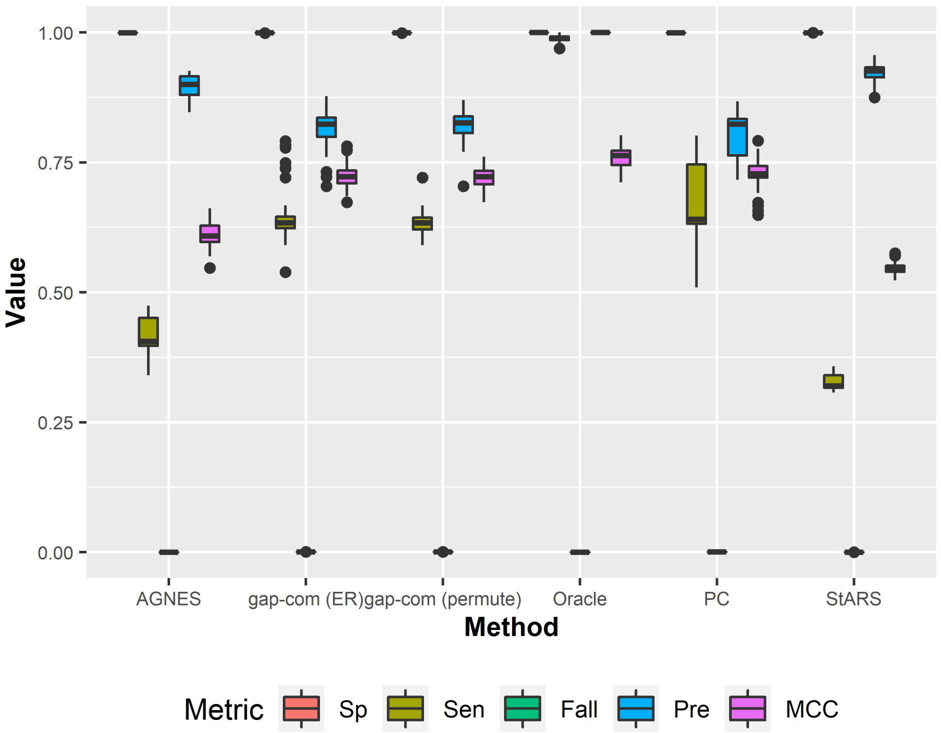

Figure 34: Hard thresholding, the **star graph model with orphan nodes**, $p = 500$, $n = 200$.

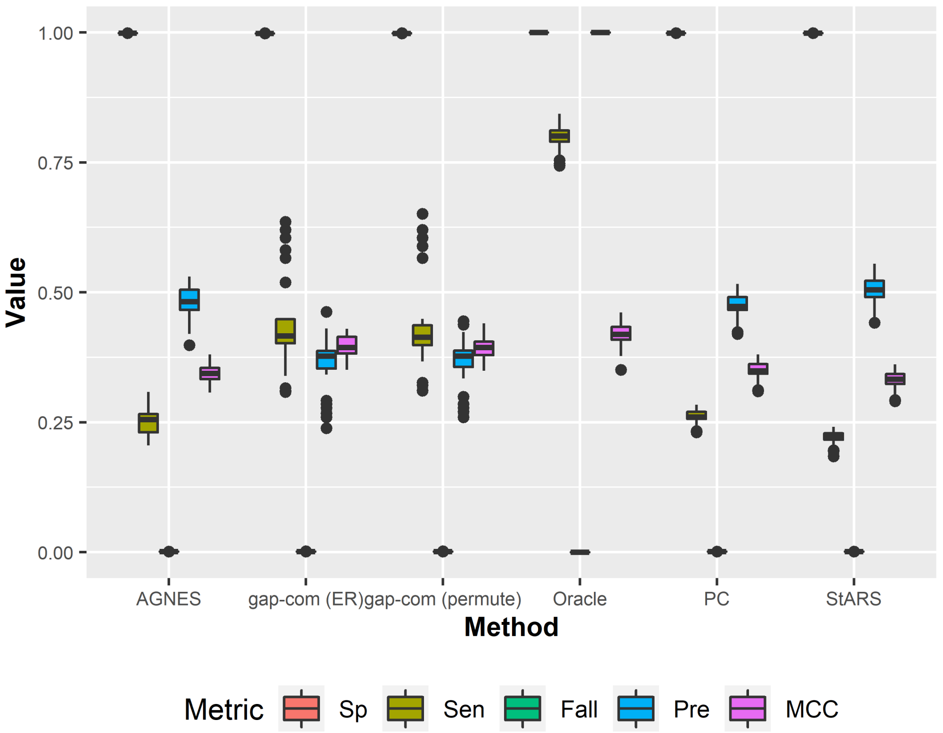

Figure 35: Hard thresholding, the **Barabási–Albert (scale-free) graph model with orphan nodes**, $p = 500$, $n = 200$.

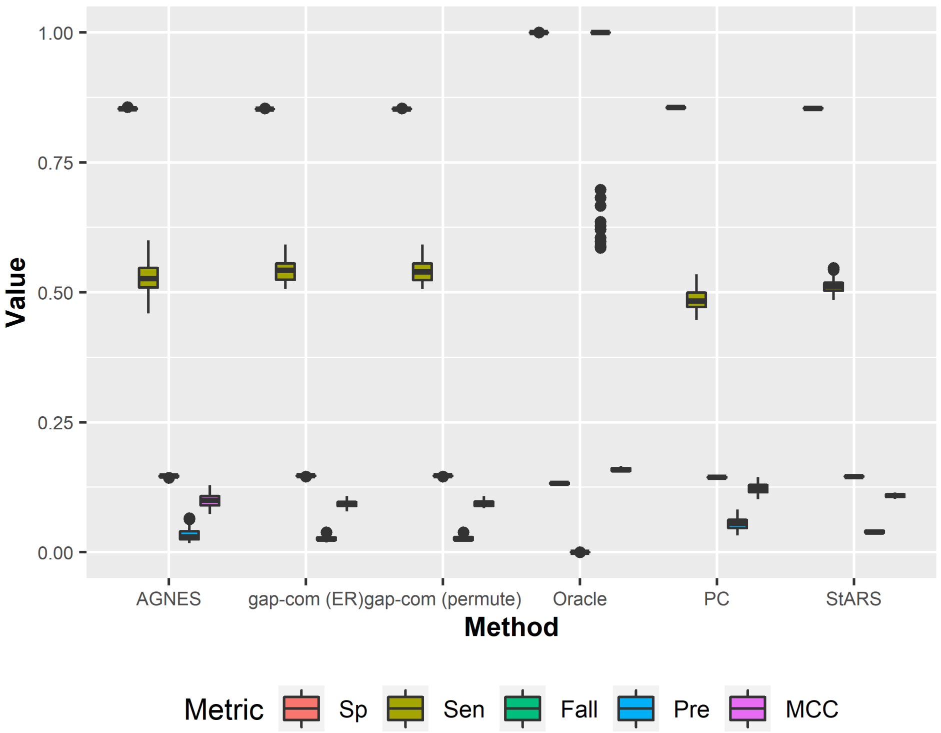

Figure 36: Hard thresholding, the **Erdos-Renyi graph model with orphan nodes**, $p = 500$, $n = 200$.

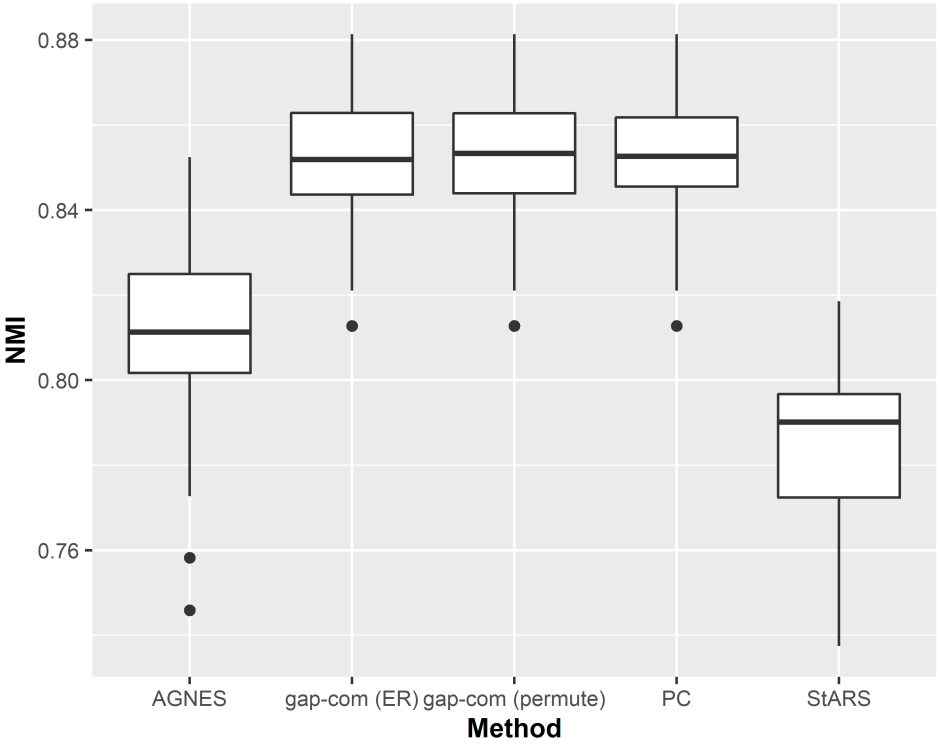

Figure 37: Hard thresholding, the **cluster graph model with orphan nodes**, $p = 500$, $n = 200$.

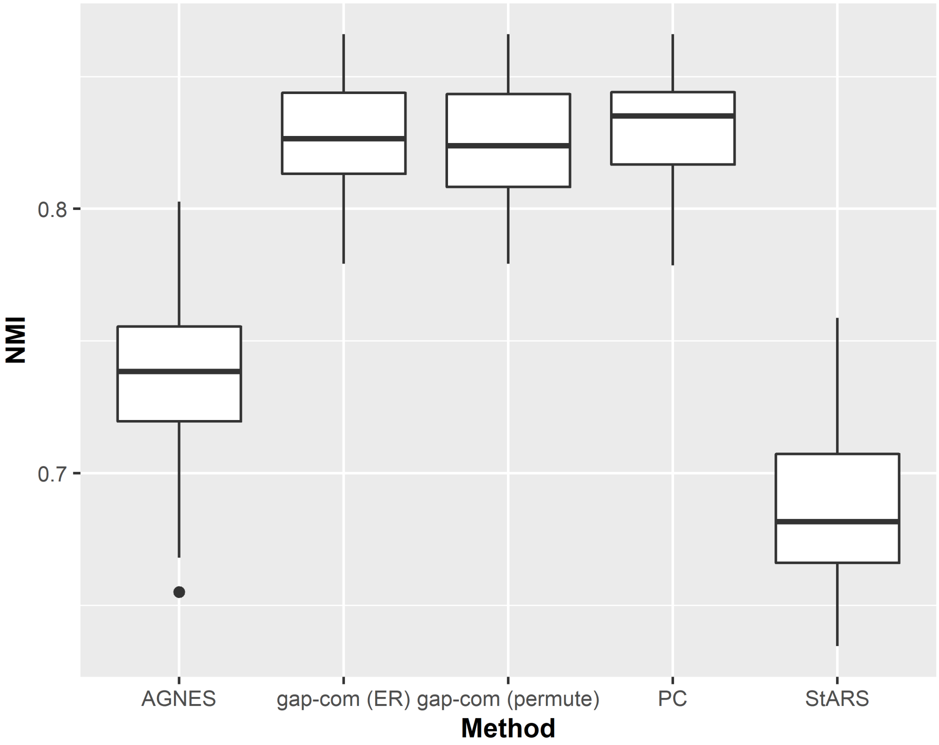

Figure 38: Hard thresholding, the **star graph model with orphan nodes**, $p = 500$, $n = 200$.

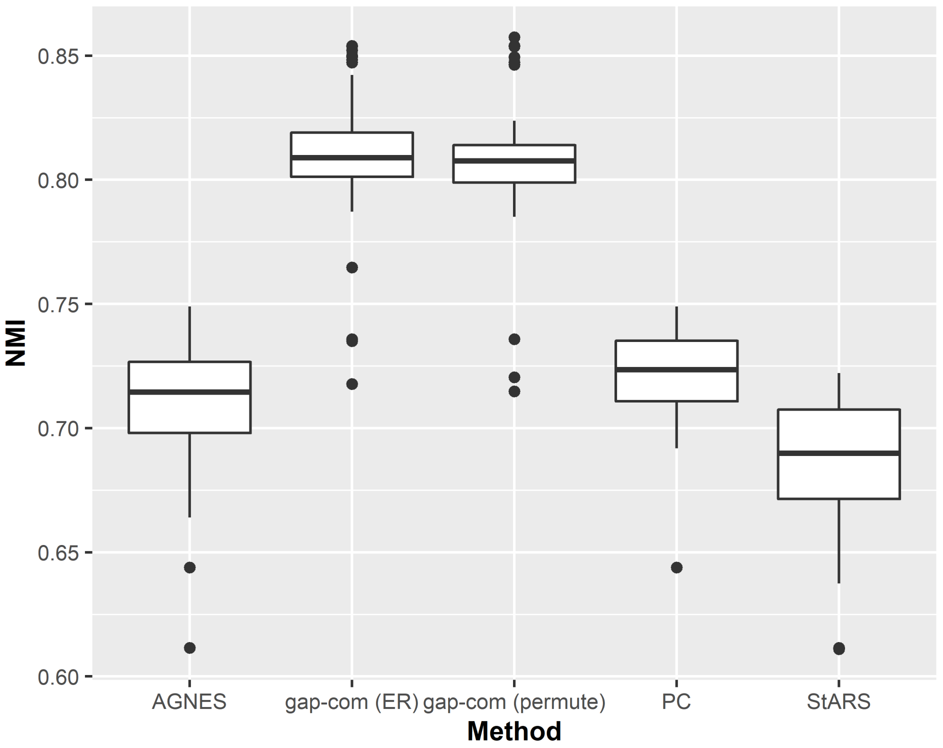

Figure 39: Hard thresholding, the **Barabási–Albert (scale-free) graph model with orphan nodes**, $p = 500$, $n = 200$.

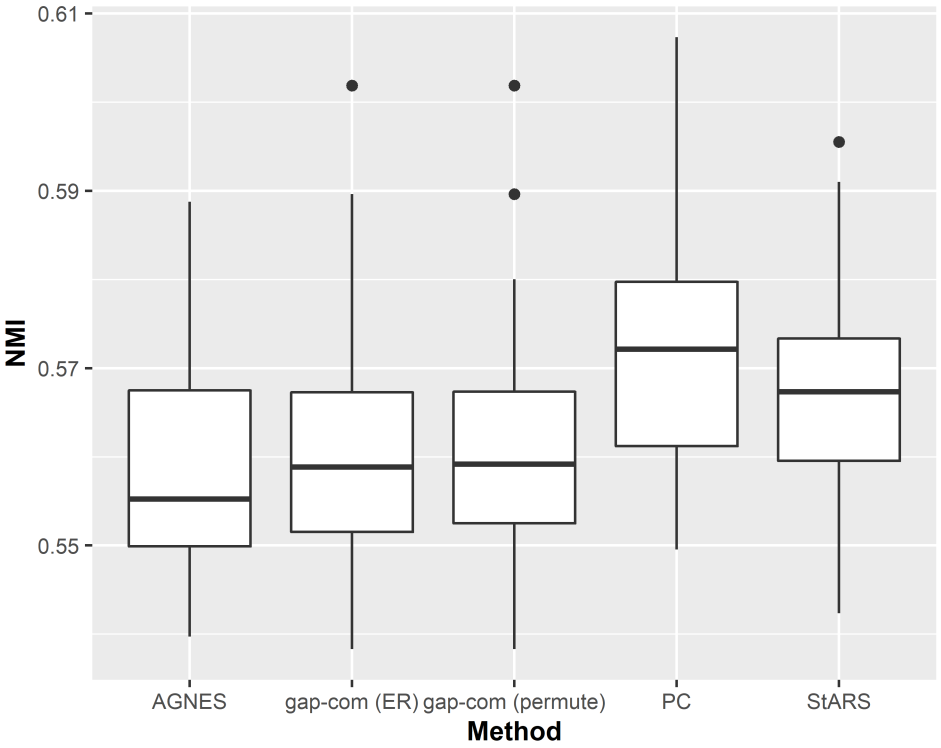

Figure 40: Hard thresholding, the **Erdos-Renyi graph model with orphan nodes**, $p = 500$, $n = 200$.

2.2 Changing the sample size

These results are averaged over 20 simulation replications.

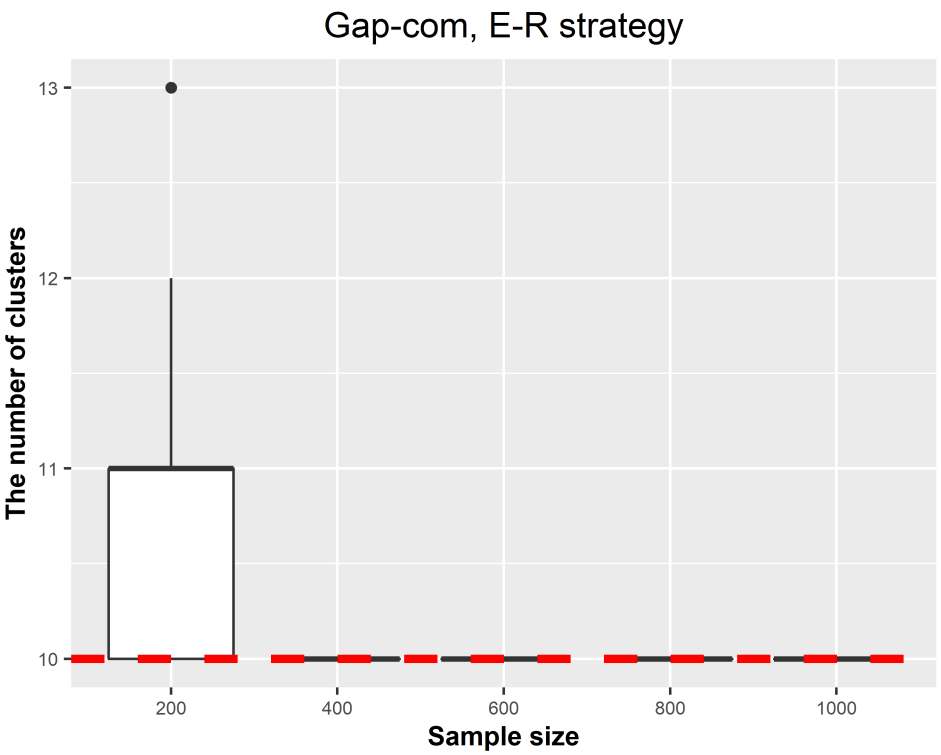

Figure 41: The number of clusters detected from the graphical model selected with gap-com E-R strategy. The graph is estimated using the hard thresholding. The data depends on the structure of the **cluster graph** model. Here $p = 200$ and the sample size is reported on the horizontal axis. The dashed horizontal line illustrates the true number of clusters.

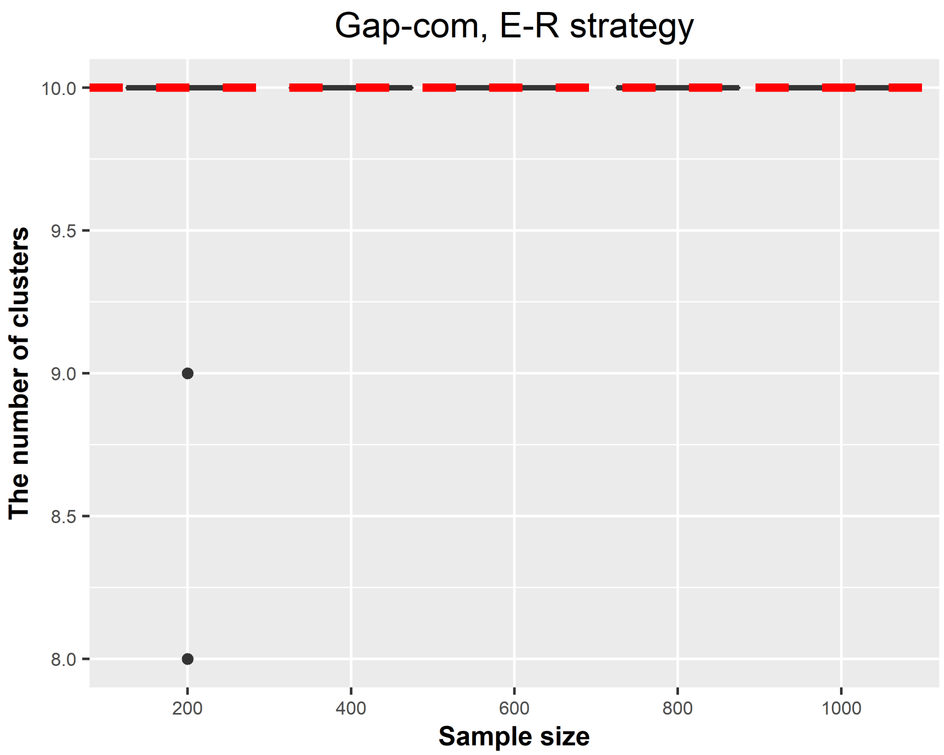

Figure 42: The number of clusters detected from the graphical model selected with gap-com E-R strategy. The graph is estimated using the hard thresholding. The data depends on the structure of the **star graph** model. Here $p = 200$ and the sample size is reported on the horizontal axis. The dashed horizontal line illustrates the true number of clusters.

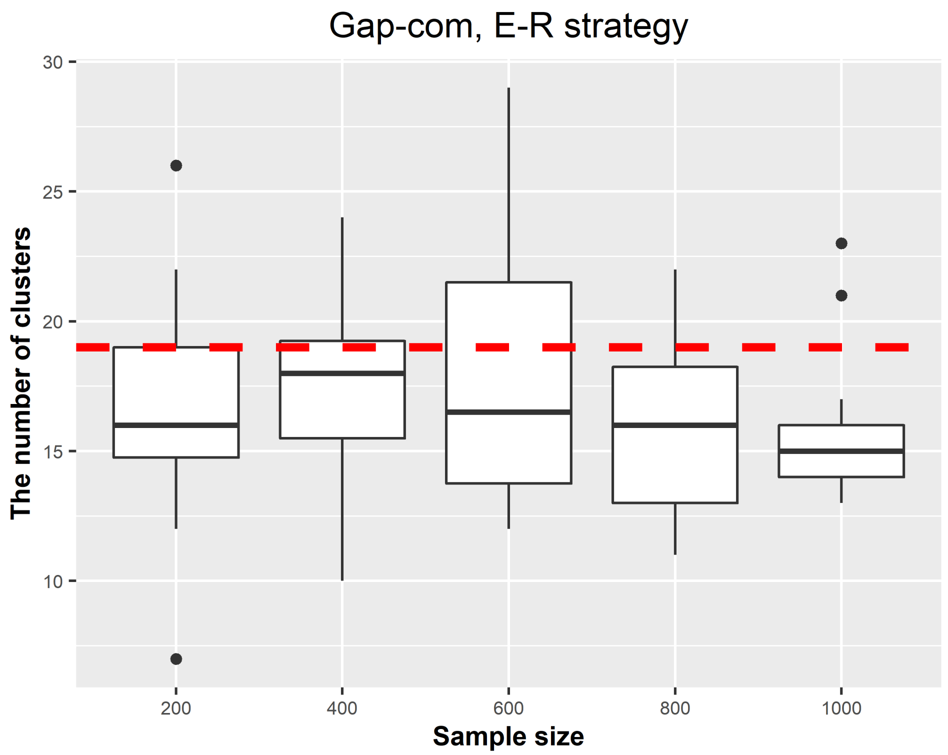

Figure 43: The number of clusters detected from the graphical model selected with gap-com E-R strategy. The graph is estimated using the hard thresholding. The data depends on the structure of the **Barabási–Albert (scale-free) graph** model. Here $p = 200$ and the sample size is reported on the horizontal axis. The dashed horizontal line illustrates the true number of clusters.

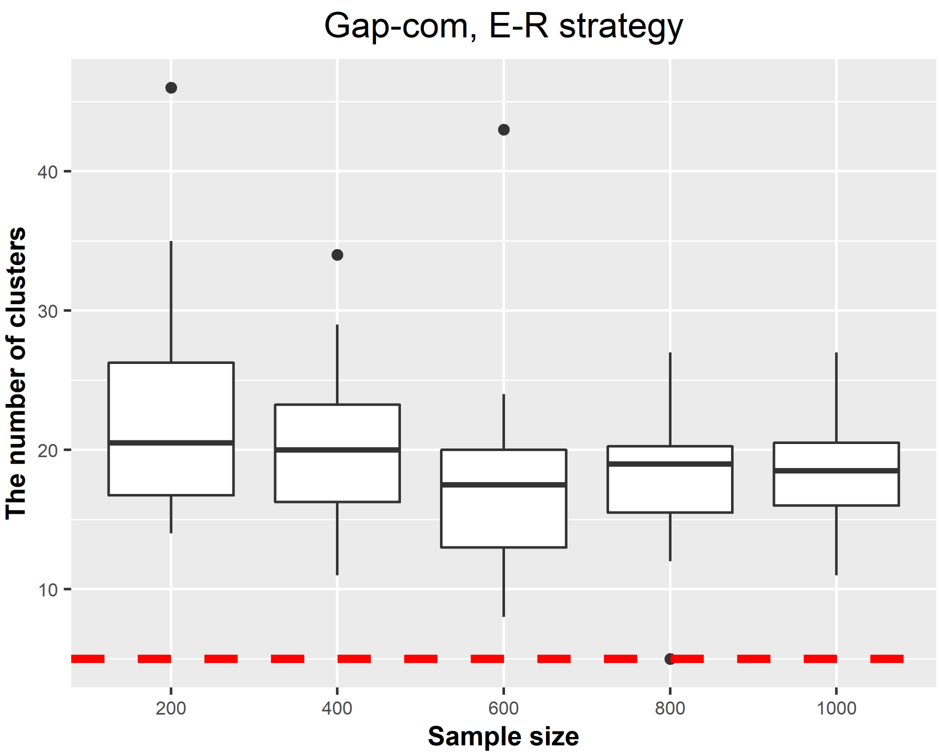

Figure 44: The number of clusters detected from the graphical model selected with gap-com E-R strategy. The graph is estimated using the hard thresholding. The data depends on the structure of the **Erdos-Renyi (random) graph** model. Here $p = 200$ and the sample size is reported on the horizontal axis. The dashed horizontal line illustrates the true number of clusters.

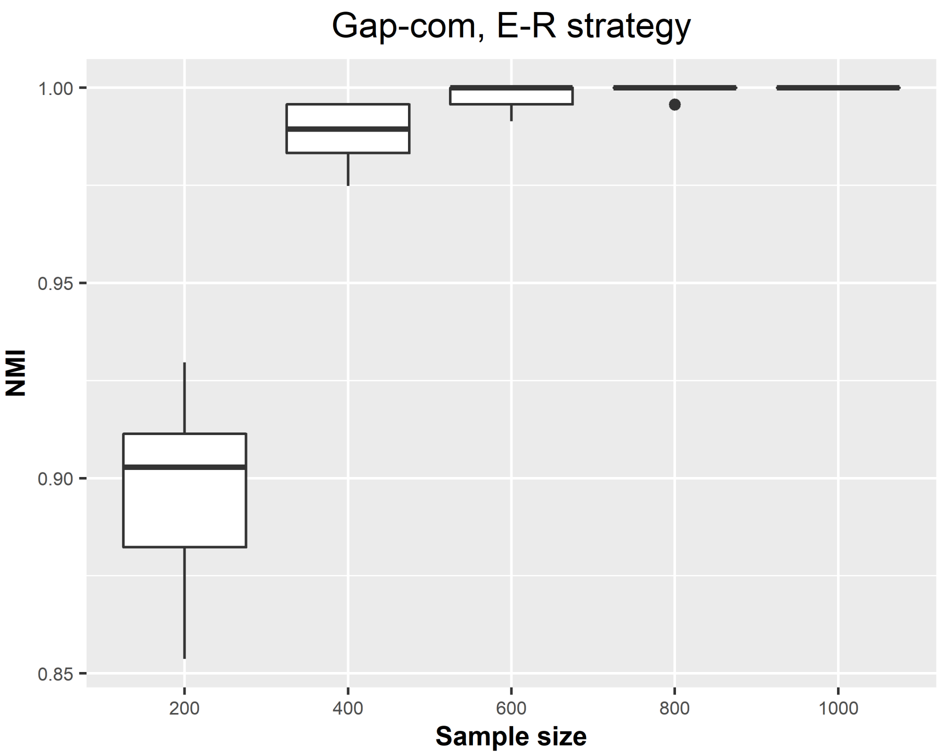

Figure 45: Normalized Mutual Information (NMI) scores when the clustering of the graphical model selected with gap-com E-R strategy is compared to the "ground truth" clustering. The graph is estimated using the hard thresholding. The data depends on the structure of the **cluster graph** model. Here $p = 200$ and the sample size is reported on the horizontal axis. The dashed horizontal line illustrates the true number of clusters.

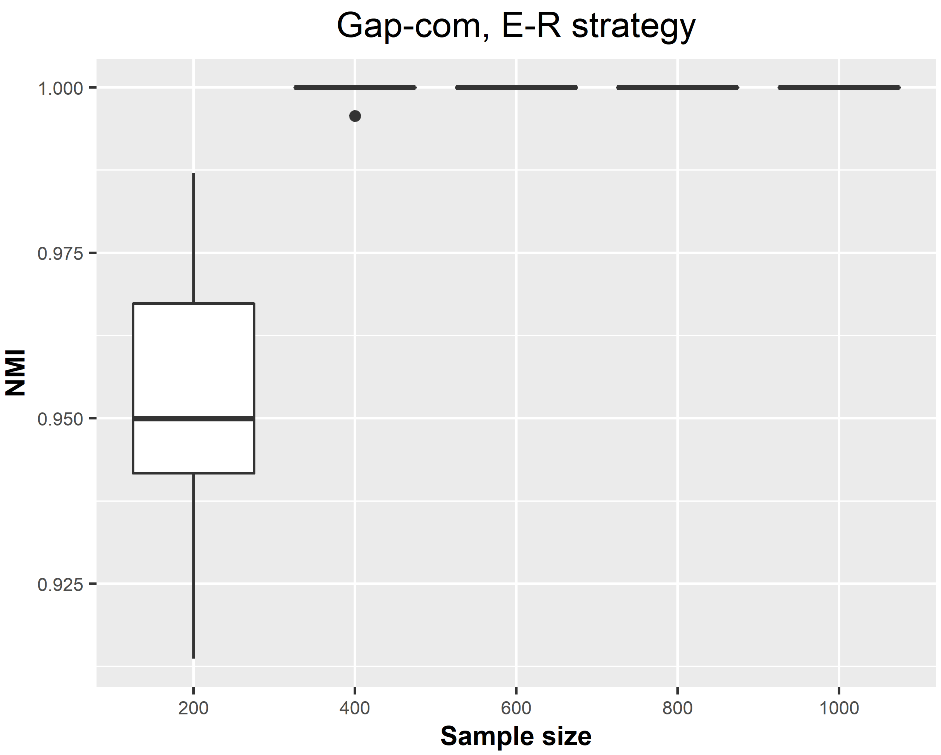

Figure 46: Normalized Mutual Information (NMI) scores when the clustering of the graphical model selected with gap-com E-R strategy is compared to the “ground truth” clustering. The graph is estimated using the hard thresholding. The data depends on the structure of the **star graph** model. Here $p = 200$ and the sample size is reported on the horizontal axis. The dashed horizontal line illustrates the true number of clusters.

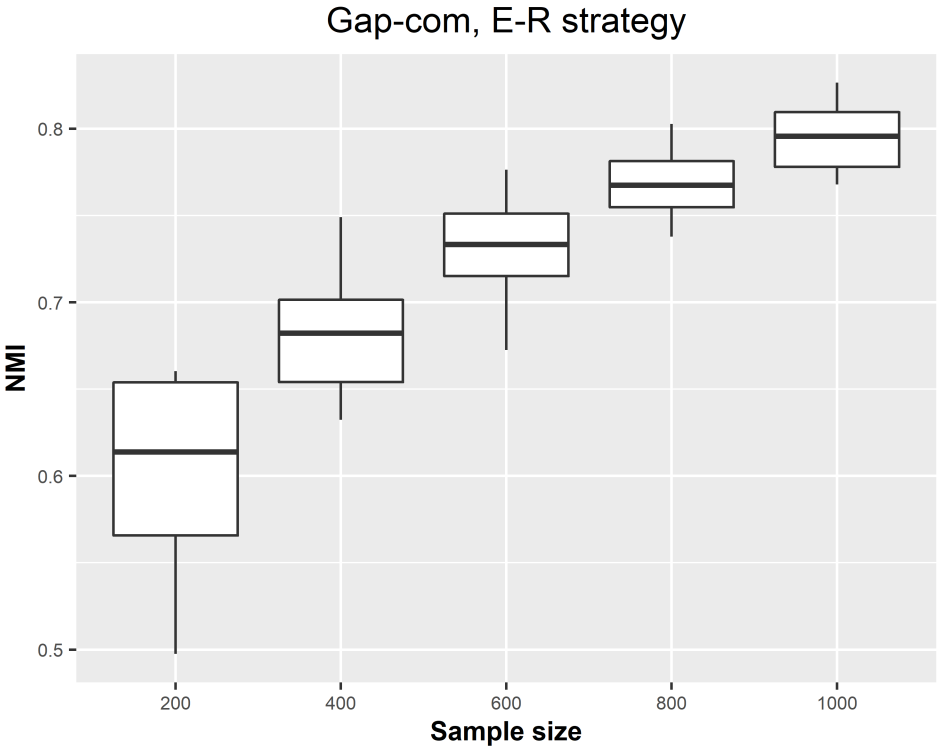

Figure 47: Normalized Mutual Information (NMI) scores when the clustering of the graphical model selected with gap-com E-R strategy is compared to the "ground truth" clustering. The graph is estimated using the hard thresholding. The data depends on the structure of the **Barabási–Albert (scale-free) graph** model. Here $p = 200$ and the sample size is reported on the horizontal axis. The dashed horizontal line illustrates the true number of clusters.

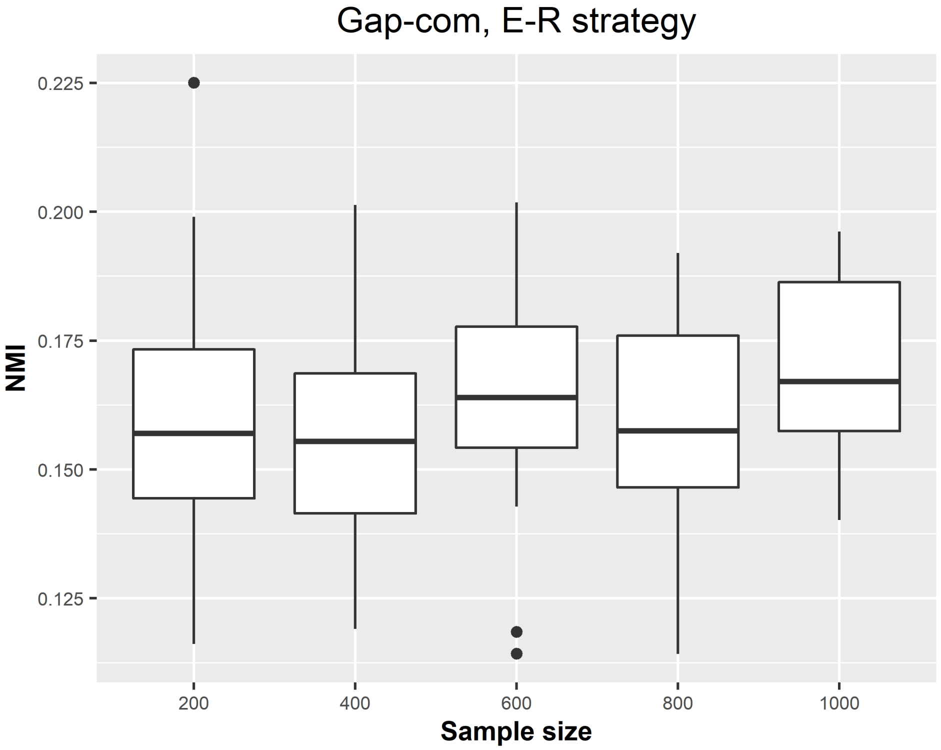

Figure 48: Normalized Mutual Information (NMI) scores when the clustering of the graphical model selected with gap-com E-R strategy is compared to the "ground truth" clustering. The graph is estimated using the hard thresholding. The data depends on the structure of the **Erdos-Renyi (random) graph** model. Here $p = 200$ and the sample size is reported on the horizontal axis. The dashed horizontal line illustrates the true number of clusters.

2.3 Using different community detection methods with gap-com

We used 50 simulation replications to examine how robust the gap-com is to the change of the community detection algorithm. We used Walktrap, Fast and greedy, and Propagating labels community detection algorithms with gap-com. All these community detection algorithms are implemented in the R-package `igraph`.

In particular, we examined how does the gap-com statistic, the value of the tuning parameter, and the NMI score change when the community

detection algorithm is changed. Here $p = 200$ and $n = 100$. Again, we only used the hard thresholding as the network estimation method and the E-R strategy with gap-com.

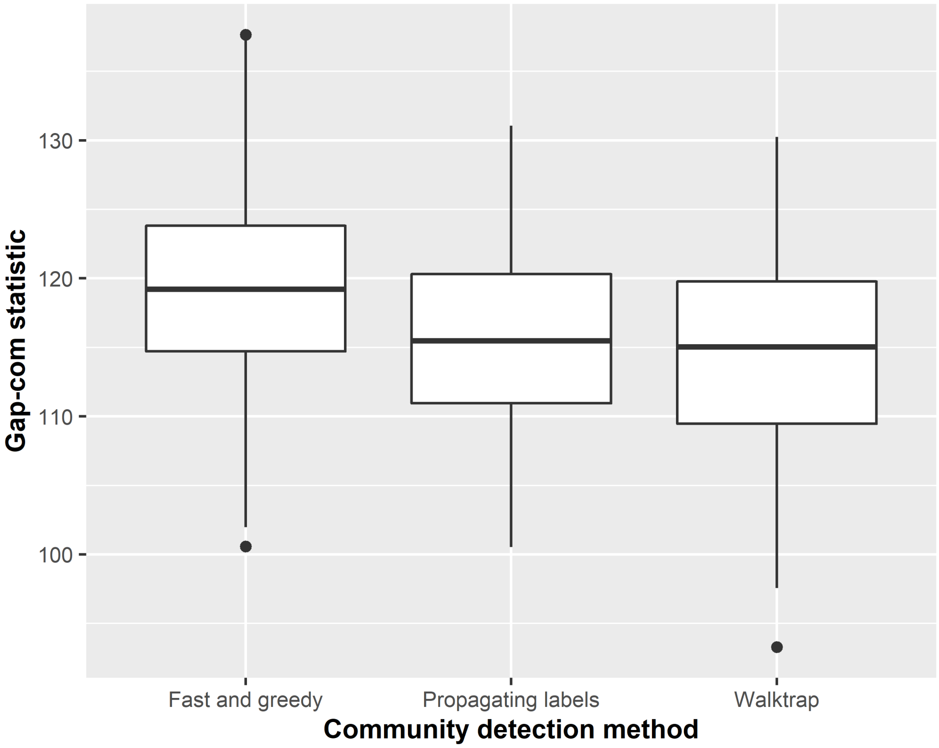

Figure 49: The gap-com statistic when the E-R strategy is used to select the graphical model. The name of the community detection method used with gap-com is reported on the horizontal axis. The graph is estimated using the hard thresholding. The data depends on the structure of the **cluster graph** model. Here $p = 200$.

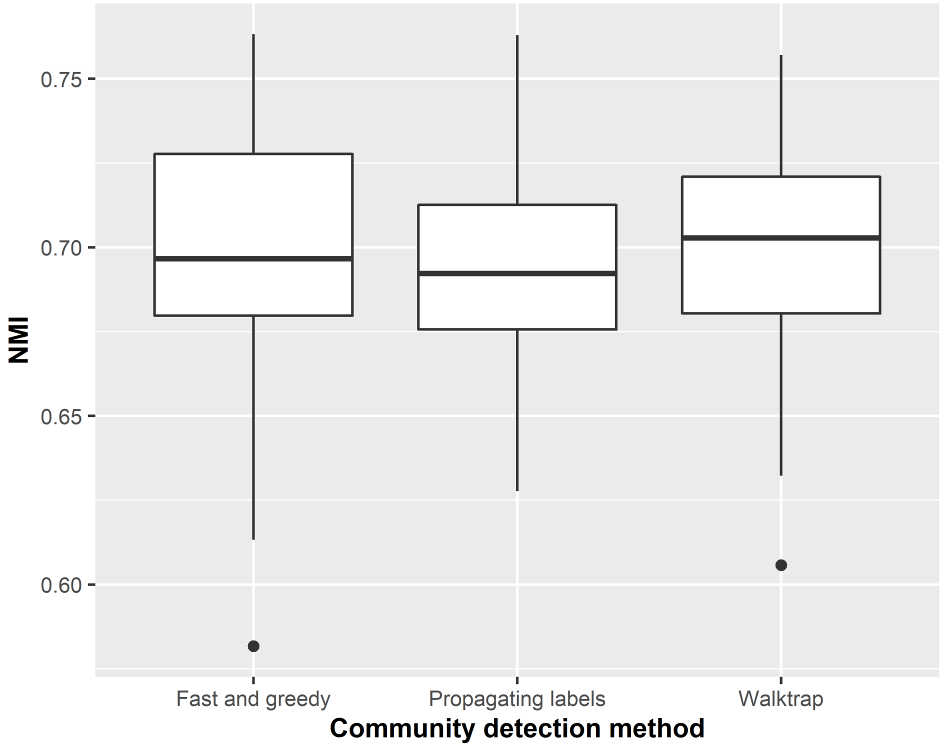

Figure 50: Normalized Mutual Information (NMI) scores when the clustering of the graphical model selected with gap-com E-R strategy is compared to the "ground truth" clustering. The name of the community detection method used with gap-com is reported on the horizontal axis. The graph is estimated using the hard thresholding. The data depends on the structure of the **cluster graph** model. Here $p = 200$.

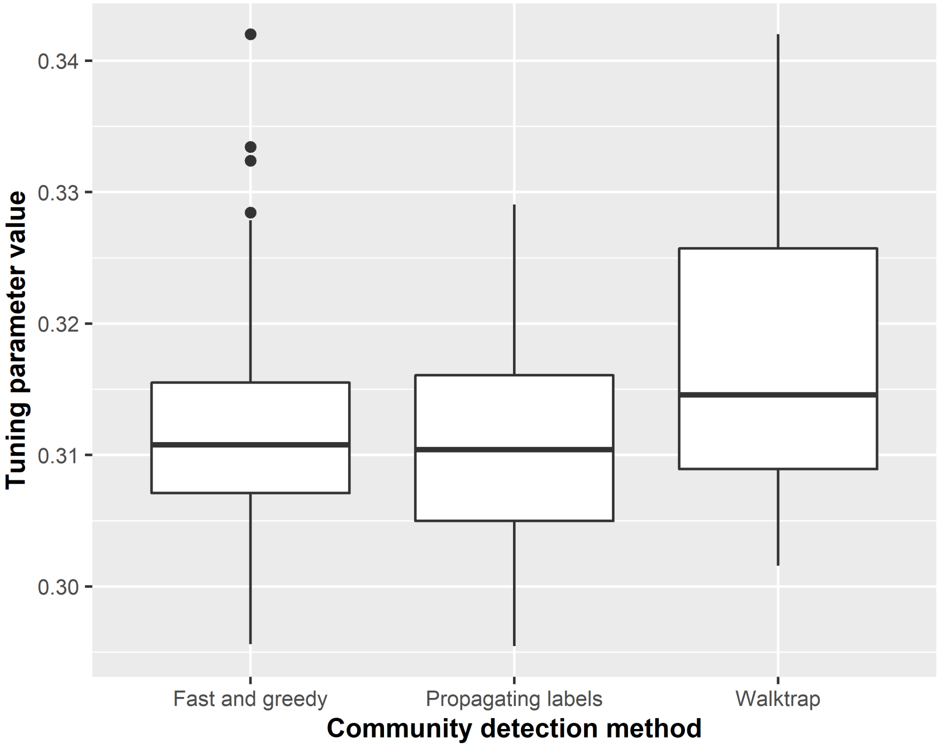

Figure 51: The value of the selected tuning parameter when the E-R strategy is used to select the graphical model. The name of the community detection method used with gap-com is reported on the horizontal axis. The graph is estimated using the hard thresholding. The data depends on the structure of the **cluster graph** model. Here $p = 200$.

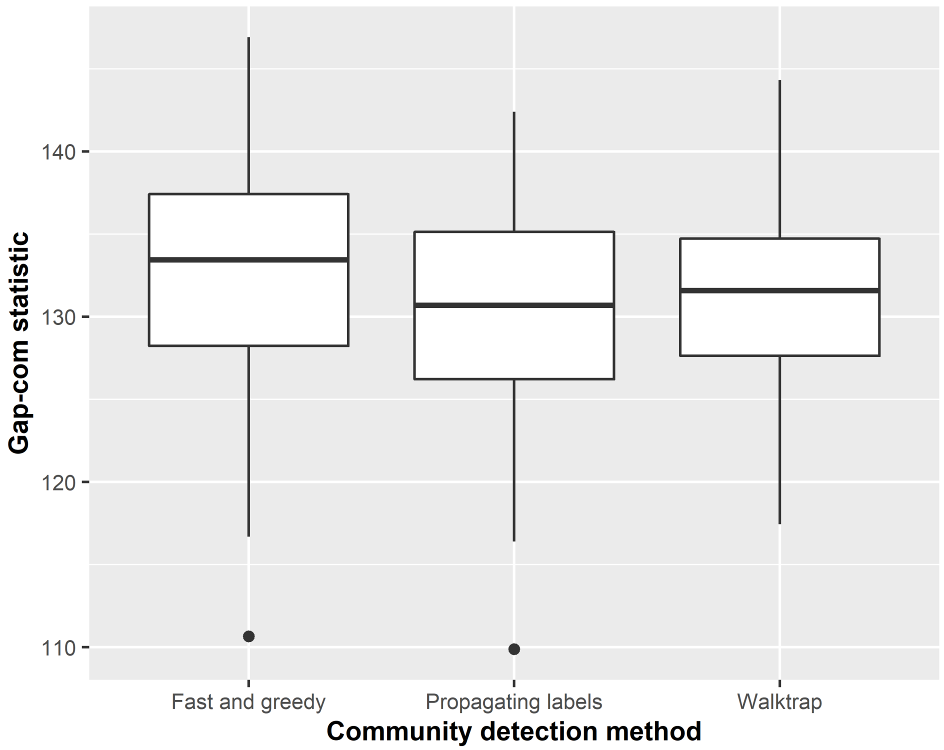

Figure 52: The gap-com statistic when the E-R strategy is used to select the graphical model. The name of the community detection method used with gap-com is reported on the horizontal axis. The graph is estimated using the hard thresholding. The data depends on the structure of the **star graph** model. Here $p = 200$.

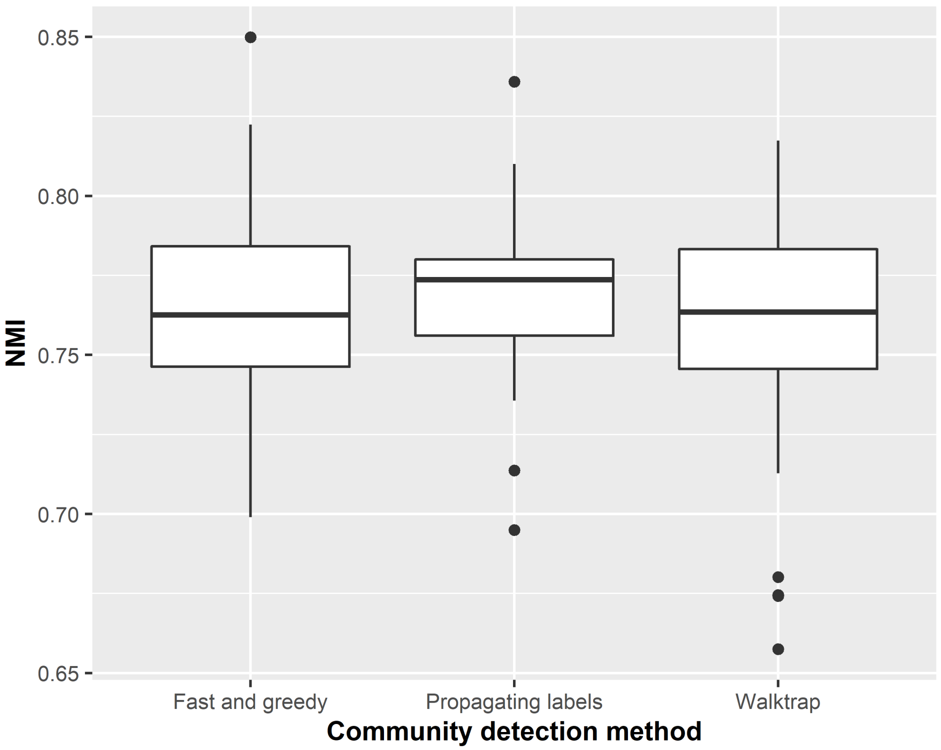

Figure 53: Normalized Mutual Information (NMI) scores when the clustering of the graphical model selected with gap-com E-R strategy is compared to the "ground truth" clustering. The name of the community detection method used with gap-com is reported on the horizontal axis. The graph is estimated using the hard thresholding. The data depends on the structure of the **star graph** model. Here $p = 200$.

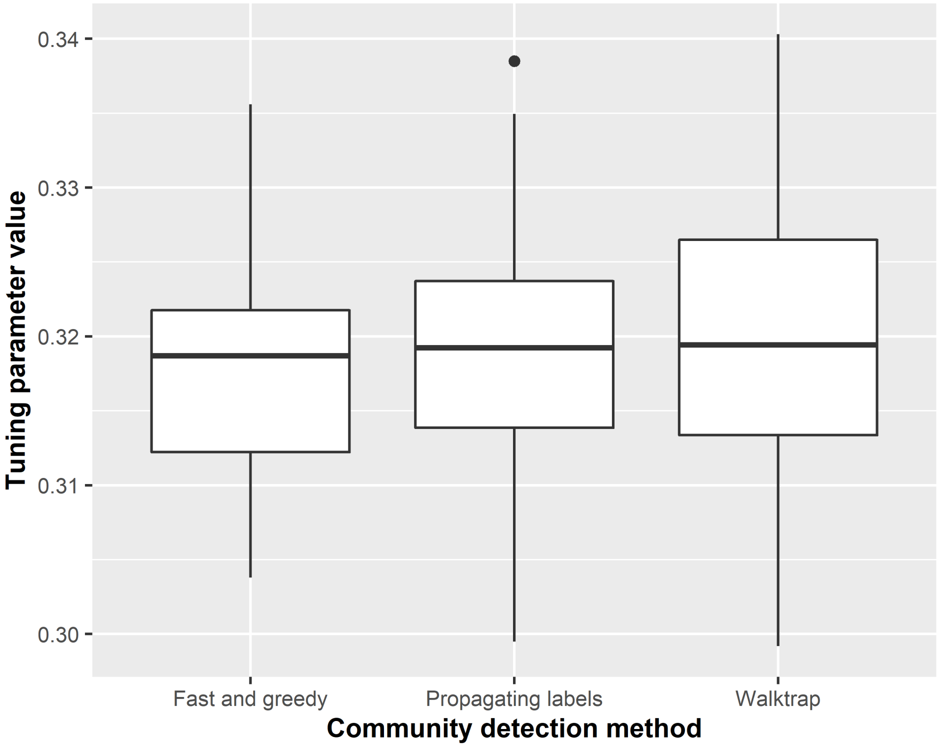

Figure 54: The value of the selected tuning parameter when the E-R strategy is used to select the graphical model. The name of the community detection method used with gap-com is reported on the horizontal axis. The graph is estimated using the hard thresholding. The data depends on the structure of the **star graph** model. Here $p = 200$.

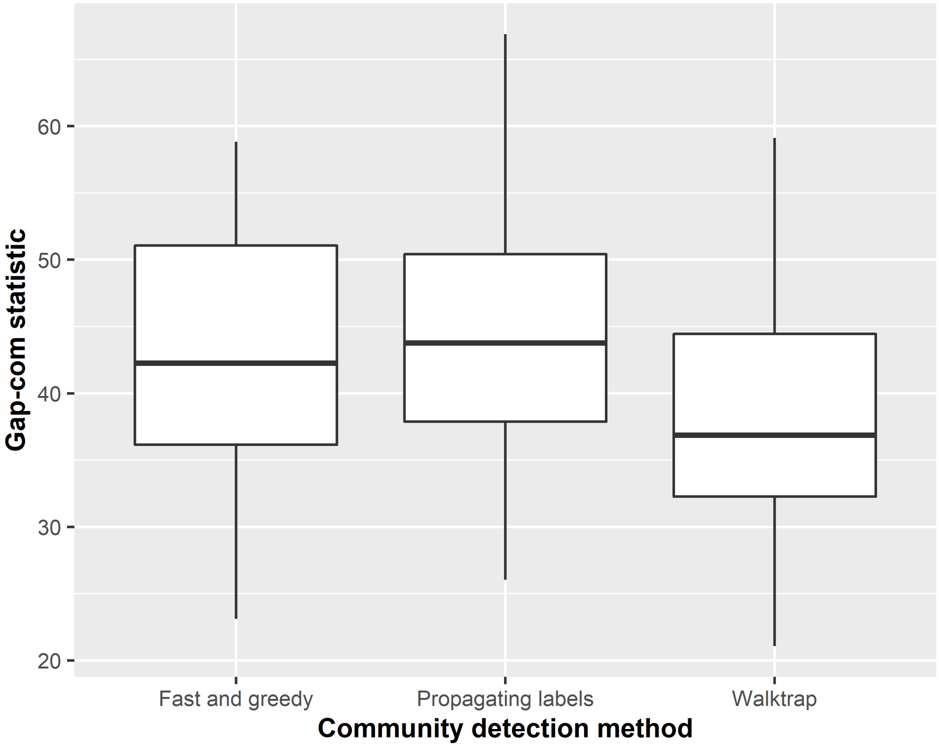

Figure 55: The gap-com statistic when the E-R strategy is used to select the graphical model. The name of the community detection method used with gap-com is reported on the horizontal axis. The graph is estimated using the hard thresholding. The data depends on the structure of the **Barabási–Albert (scale-free) graph** model. Here $p = 200$.

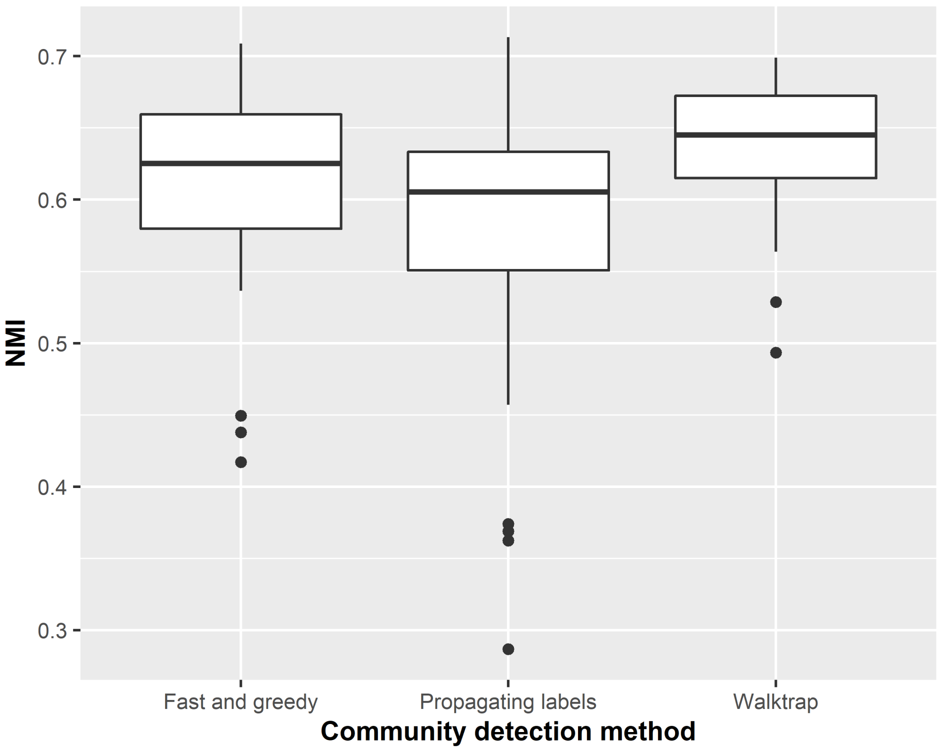

Figure 56: Normalized Mutual Information (NMI) scores when the clustering of the graphical model selected with gap-com E-R strategy is compared to the "ground truth" clustering. The name of the community detection method used with gap-com is reported on the horizontal axis. The graph is estimated using the hard thresholding. The data depends on the structure of the **Barabási–Albert (scale-free) (random) graph** model. Here $p = 200$.

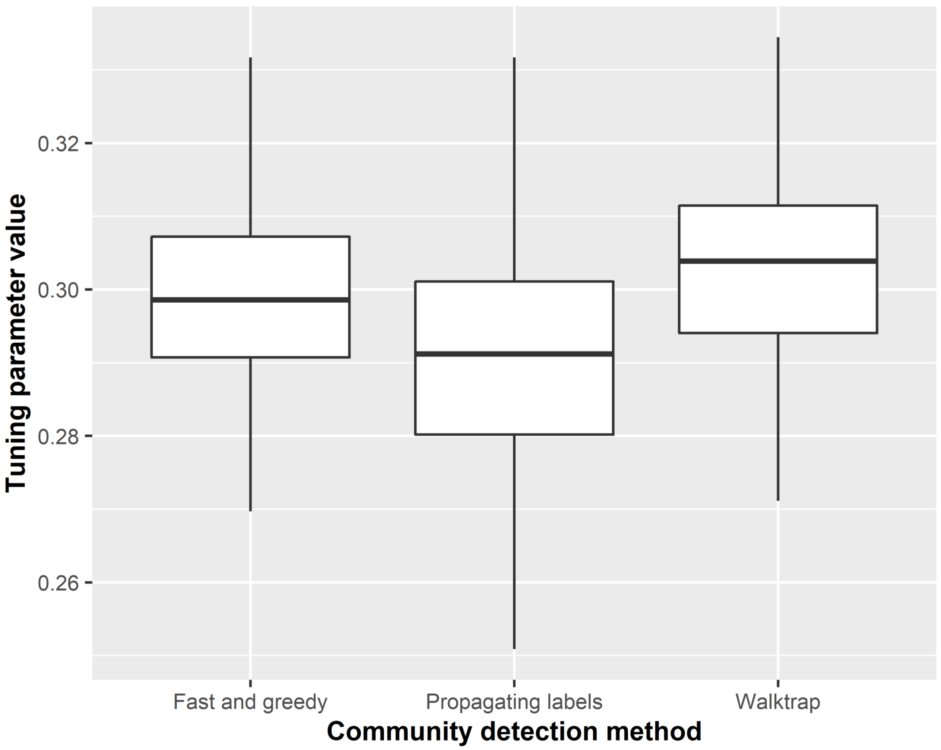

Figure 57: The value of the selected tuning parameter when the E-R strategy is used to select the graphical model. The name of the community detection method used with gap-com is reported on the horizontal axis. The graph is estimated using the hard thresholding. The data depends on the structure of the **Barabási–Albert (scale-free) graph** model. Here $p = 200$.

3 *S.aureus* DREAM5 data – Pathogen module of the co-expression network

We analysed the gene-expression data set of a human pathogen *Staphylococcus aureus* (hereafter *S. aureus*) used previously in the DREAM5 (Dialogue on Reverse Engineering Assessment and Methods) network inference challenge (Marbach et al., 2012). Overall there are expression levels of 2810 genes for 160 samples. From these 2810 genes, about 5% are decoy genes which were introduced by randomly selecting gene expression values from the compendium, itself (see supplementary Note 1 of (Marbach et al., 2012)). Genes

were anonymized for the original DREAM5 challenge but true gene IDs are available at the DREAM Challenges homepage.

The co-expression networks were computed by hard-threholding pairwise correlation coefficients. Overall, we used 50 different hard-threshold cutoff values in the co-expression network inference determined with the R-package `huge`.

Here are small subgraphs which corresponds to a module of 27 genes identified in (Marbach et al., 2012) that is highly enriched for pathogenic genes.

[!p]

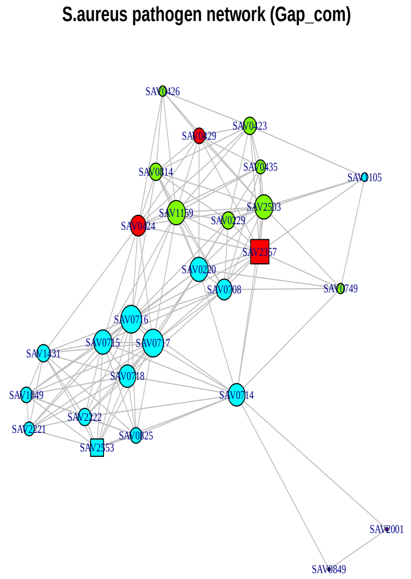

Figure 58: A small subgraph from the *S.aureus* co-expression network selected with gap-com. Nodes correspond to the genes from the module of 27 genes identified in Marbach et al. (2012) that is highly enriched for pathogenic genes. Transcription factors are shaped as squares. Different colors correspond to different communities in the larger network identified with the Walktrap algorithm. Node sizes are relative to the node degree.

[!p]

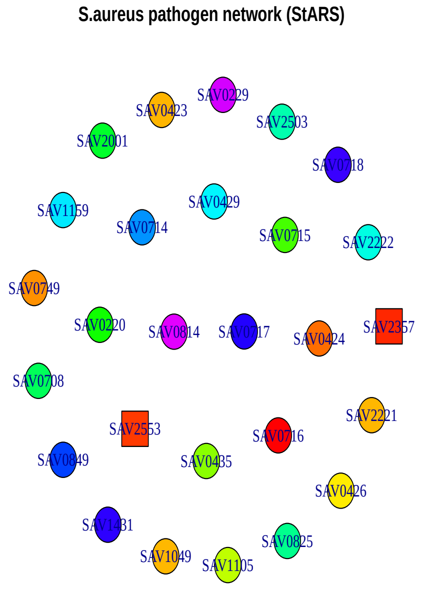

Figure 59: A small subgraph from the *S.aureus* co-expression network selected with StARS. Nodes correspond to the genes from the module of 27 genes identified in Marbach et al. (2012) that is highly enriched for pathogenic genes. Transcription factors are shaped as squares. Different colors correspond to different communities in the larger network identified with the Walktrap algorithm. Node sizes are relative to the node degree.

[!p]

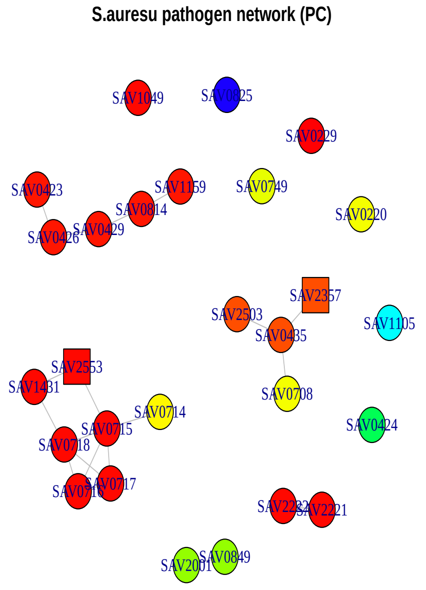

Figure 60: A small subgraph from the *S.aureus* co-expression network selected with the path connectivity (PC) criterion. Nodes correspond to the genes from the module of 27 genes identified in Marbach et al. (2012) that is highly enriched for pathogenic genes. Transcription factors are shaped as squares. Different colors correspond to different communities in the larger network identified with the Walktrap algorithm. Node sizes are relative to the node degree.

[!p]

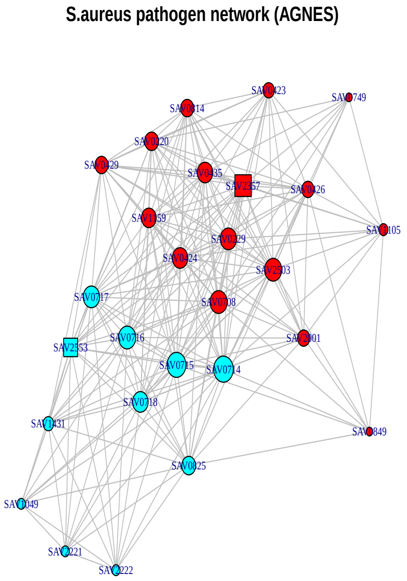

Figure 61: A small subgraph from the *S.aureus* co-expression network selected with the AGglomerative NESting (AGNES) algorithm. Nodes correspond to the genes from the module of 27 genes identified in Marbach et al. (2012) that is highly enriched for pathogenic genes. Transcription factors are shaped as squares. Different colors correspond to different communities in the larger network identified with the Walktrap algorithm. Node sizes are relative to the node degree.

References

Hsieh, C.-J., Sustik, M. A., Dhillon, I. S., Ravikumar, P. K., and Poldrack, R. (2013). BIG & QUIC: Sparse inverse covariance estimation for a million variables. In Burges, C. J. C., Bottou, L., Welling, M., Ghahramani, Z., and Weinberger, K. Q., editors, *Advances in Neural Information Processing Systems 26*, pages 3165–3173. Curran Associates, Inc.

Marbach, D., Costello, J. C., Küffner, R., Vega, N. M., Prill, R. J., Camacho, D. M., Allison, K. R., Aderhold, A., Bonneau, R., Chen, Y., et al. (2012). Wisdom of crowds for robust gene network inference. *Nature Methods*, 9:796–803.
